# Phytochemical Quorum-Sensing Inhibitors Against Bacterial Pathogens: Mechanisms of Action and Translational Challenges

**DOI:** 10.3390/cimb48020214

**Published:** 2026-02-14

**Authors:** Christos Papaneophytou

**Affiliations:** Department of Life Sciences, School of Life and Health Sciences, University of Nicosia, Nicosia 2417, Cyprus; papaneophytou.c@unic.ac.cy; Tel.: +357-22841941

**Keywords:** quorum sensing, autoinducers, phytochemicals, microbiota, bacterial virulence, anti-virulence strategies

## Abstract

Antimicrobial resistance is a critical global health challenge, driven by the rapid emergence of multidrug-resistant bacterial pathogens and exacerbated by extensive antibiotic use, which imposes intense selective pressure and disrupts host-associated microbial communities. In this context, quorum sensing (QS), a conserved molecular communication system that coordinates population-level gene regulation, virulence expression, and biofilm development, has emerged as an attractive target for anti-virulence intervention. A growing body of evidence indicates that phytochemicals, such as curcumin, carvacrol, carnosol, eugenol, and chlorogenic acid, can modulate key QS pathways, including acyl-homoserine lactone-, autoinducing peptide-, and LuxS/AI-2-mediated signaling, thereby attenuating pathogenic behaviors at sub-inhibitory concentrations that do not directly impair bacterial viability. Despite this promise, the translational development of phytochemical-based QS inhibitors remains limited. Because QS also regulates cooperative and homeostatic functions in beneficial bacteria, QS-targeted interventions raise concerns about microbiome disruption and ecological imbalance. Furthermore, the literature is marked by substantial methodological heterogeneity, reliance on indirect phenotypic endpoints, limited molecular target validation, and insufficient assessment of toxicity, bioavailability, and pharmacokinetics. The predominance of simplified in vitro models further constrains extrapolation to complex host-associated and polymicrobial environments. This review critically examines the molecular mechanisms underlying phytochemical modulation of bacterial QS, synthesizes pathogen-focused experimental evidence, and evaluates key translational challenges arising from QS conservation, microbiome considerations, and methodological limitations. Addressing these barriers through mechanism-resolved experimentation, standardized evaluation frameworks, and microbiome-aware testing strategies will be essential for advancing phytochemical QS inhibitors toward clinically and industrially relevant anti-virulence applications.

## 1. Introduction

Antibiotic resistance (AR) has become one of the most urgent global health challenges, undermining the long-term success of standard antimicrobial treatments. AR arises when bacteria acquire or regulate genetic and phenotypic traits that reduce susceptibility to antimicrobial agents, thereby diminishing the effectiveness of conventional therapies [1]. Beyond classical genetic resistance mechanisms, regulatory processes that promote antimicrobial tolerance and persistence are increasingly recognized as essential contributors to treatment failure. Among these, bacterial quorum sensing (QS), a cell-to-cell communication system that coordinates virulence, biofilm formation, stress responses, and horizontal gene transfer, has emerged as a key regulatory network that indirectly supports the persistence of resistant infections [2]. In this context, phytochemicals have attracted growing interest as QS inhibitors (QSIs) that attenuate QS-regulated pathogenic behaviors without imposing direct bactericidal pressure. QS is a conserved communication mechanism that enables bacteria to coordinate collective behaviors in response to population density and community context [3]. It relies on the production, release, accumulation, and detection of small signaling molecules termed autoinducers (AIs). As bacterial numbers increase, AI concentrations rise and, once a threshold is reached, trigger coordinated activation or repression of QS-regulated genes across the community [4,5]. Through QS, bacteria integrate information about local population structure with environmental and ecological cues, enabling dynamic adjustment of physiological programs within multispecies communities [6]. QS-regulated behaviors are often inefficient or ineffective at the single-cell level but become advantageous when executed collectively, enabling coordinated processes such as biofilm maturation, motility, conjugative gene transfer, and regulated expression of virulence-associated functions [7]. This population-level coordination underscores the social organization of bacterial communities and challenges the traditional view of bacteria as strictly unicellular organisms [8]. Despite diversity in signal chemistries and regulatory architectures, QS systems share a common operational logic: extracellular AIs accumulate in a cell-density–dependent manner and are detected by cognate cytoplasmic or membrane-associated receptors, which initiate signal-transduction cascades and frequently engage positive feedback (autoinduction) loops that synchronize gene expression at the community level [9,10].

QS plays a central role in bacterial pathogenicity by coordinating the timing and magnitude of virulence factor expression, thereby optimizing infection success against host defenses [11]. Consistent with this broad regulatory scope, QS has been estimated to influence approximately 4–10% of the bacterial genome and more than 20% of the proteome [12,13]. In addition to regulating virulence and biofilm programs, QS indirectly contributes to antimicrobial treatment failure by shaping phenotypes associated with tolerance and resistance [14]. Mechanistically, QS interfaces with multidrug efflux regulation (e.g., MexAB-OprM in *Pseudomonas aeruginosa*), stress-response pathways (including oxidative and SOS responses), and persistence-associated states [15,16], and it can facilitate the dissemination of resistance determinants by promoting horizontal gene transfer through conjugation and plasmid mobilization [17,18]. Together, these interconnected outputs position QS as a central regulator of pathogenicity and adaptive resistance.

Given its central regulatory role in pathogenic bacteria, QS has emerged as an attractive target for anti-virulence strategies that attenuate pathogenic traits without imposing direct bactericidal pressure, thereby reducing the selective forces that drive resistance [19]. In this context, natural products, including phytochemicals, microbial metabolites, and marine-derived compounds, have attracted growing interest as sources of QSIs (reviewed in [20]). Among them, phytochemicals have attracted special attention as QSIs because they have been reported to modulate QS signaling pathways and reduce virulence, biofilm formation, and infection-associated resistance phenotypes across diverse bacterial pathogens [20,21,22]. Plant-derived QS modulators, such as rosmarinic acid, apigenin, allicin, quercetin, and related compounds, have been shown to suppress QS-regulated behaviors, often at subinhibitory concentrations that do not directly inhibit bacterial growth or viability [23,24,25].

Importantly, QS-mediated communication is not limited to pathogens but is also widely used by commensal and beneficial members of the human microbiota, including lactic acid bacteria (LAB) commonly used as probiotics [26]. In these organisms, QS regulates functions associated with colonization and community stability, including adhesion, biofilm formation, metabolite production, stress tolerance, and host signaling. In addition, probiotic bacteria may produce quorum quenching (QQ) molecules that interfere with pathogen QS, thereby contributing to microbial community homeostasis [27]. This creates a key paradox for QS-targeted interventions: while QS inhibition can attenuate virulence and biofilm formation in pathogens, QS signaling may also support beneficial microbiota functions required for stable host–microbe homeostasis.

Because QS signalling logic is conserved across pathogenic and beneficial bacteria, a central unresolved question is how QS-targeting agents, particularly phytochemicals, modulate QS-regulated functions across diverse bacterial lifestyles. Although substantial evidence indicates that phytochemicals can attenuate QS-dependent virulence and biofilm formation in pathogens, the literature remains heavily skewed toward pathogen-focused studies, with comparatively few investigations examining QS modulation in commensal, symbiotic, or probiotic bacteria. Consequently, microbiome-level outcomes of QS-targeted interventions are still poorly defined, raising important safety and translational concerns. Addressing this knowledge gap is essential for evaluating the feasibility, selectivity, and clinical relevance of QS-based anti-virulence strategies. Accordingly, this review critically examines the molecular mechanisms, experimental evidence, and translational challenges associated with phytochemical modulation of bacterial quorum sensing, with particular emphasis on pathogen control, QS system conservation, and microbiome-related considerations. Although several recent excellent reviews (see, for example [4,28]) have comprehensively cataloged phytochemicals with QSIs and anti-biofilm activity, this review deliberately moves beyond compound-centric inventories. Instead, it focuses on the mechanistic ambiguities, methodological inconsistencies, and translational barriers that currently limit the development of phytochemical QS inhibitors, thereby framing QS modulation as a system.

## 2. Methodology

This narrative review was conducted through a structured qualitative analysis of the literature addressing QS mechanisms, QS inhibition, and QS modulation by natural products, with particular emphasis on bacterial pathogens, microbiome-related implications, and unresolved mechanistic and translational challenges. A comprehensive literature search was performed using PubMed, Scopus, and Web of Science.

Search strategies employed combinations of keywords including “quorum sensing,” “quorum sensing inhibition,” “quorum quenching,” “natural products,” “phytochemicals,” “anti-virulence,” “biofilm inhibition,” “antimicrobial resistance,” “LuxS/AI-2,” “AHL,” “AIP,” “microbiota,” and “probiotics.” The primary focus was on peer-reviewed articles published up to January 2026. Seminal studies published outside this timeframe were selectively included when they provided foundational mechanistic insights essential for contextual understanding of QS biology.

Studies were selected for relevance to QS regulation and QS-dependent phenotypes, including virulence, biofilm formation, and resistance-associated behaviors, and for the presence of experimental evidence supporting modulation of QS pathways by natural products. Both in vitro and in vivo studies were considered. Reviews, mechanistic investigations, and experimental reports describing phytochemical-derived QS modulators were included for synthesis and critical comparison. Articles focused exclusively on synthetic QS inhibitors without relevance to natural products or lacking experimental validation were excluded.

To ensure interpretability and reproducibility, priority was given to studies that used QS-specific readouts and provided sufficient methodological detail. Reports were deprioritized or excluded when conclusions relied primarily on nonspecific phenotypic endpoints (e.g., growth inhibition without QS assays), lacked appropriate controls or statistical reporting, used non-physiological concentrations without justification, or provided insufficient methodological detail to support evaluation of reproducibility and relevance to the review’s central themes.

Data were synthesized thematically and discussed in an integrated manner. Sections were organized by QS system type, biological context, and the mechanistic mode of QS modulation. This framework enabled critical comparisons across studies, the identification of consistent trends and contradictions, and the highlighting of unresolved questions and research challenges relevant to the development of microbiome-aware, QS-based anti-virulence strategies.

## 3. Quorum-Sensing Communication in Bacteria: Diversity of Chemical Languages

**Figure 1:**
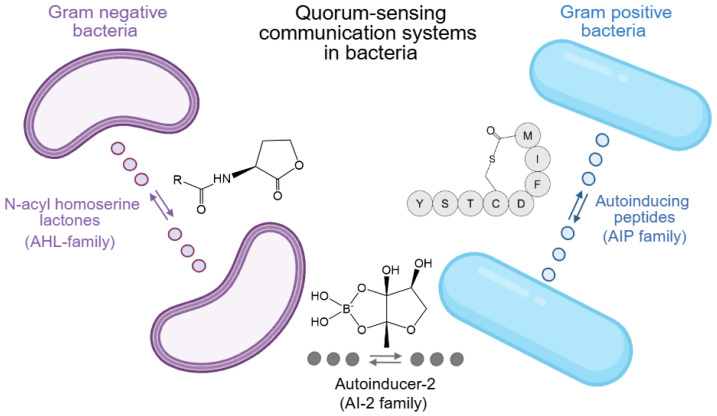
Quorum-sensing communication systems in Gram-negative and Gram-positive bacteria. Schematic overview of the major quorum-sensing (QS) signaling modalities used by bacteria to coordinate population-level behaviors. Gram-negative bacteria predominantly communicate via diffusible N-acyl homoserine lactones (AHLs), whereas Gram-positive bacteria rely primarily on secreted autoinducing peptides (AIPs) detected by membrane-associated receptors. In addition to these group-specific systems, both Gram-negative and Gram-positive bacteria utilize the LuxS-derived autoinducer-2 (AI-2) signaling pathway, which mediates intra- and interspecies communication within mixed microbial communities. Dots represent diffusible signaling molecules, arrows indicate bidirectional signal exchange, and simplified chemical motifs illustrate representative signal families. Created in BioRender. Papaneophytou, C. (2026) https://BioRender.com/hgt70kr.

As bacterial populations span diverse taxa and ecological niches, a fundamental question arises: do all bacteria communicate using the same chemical “language,” or do QS mechanisms differ fundamentally between Gram-positive (G^+^) and Gram-negative (G^−^) organisms? Although G^+^ and G^−^ bacteria employ distinct signal chemistries and signal-transduction architectures, most notably autoinducing peptides (AIPs) and acyl-homoserine lactones (AHLs), respectively, they nevertheless rely on shared organizational principles that enable population-density–dependent coordination of collective behaviors. In addition to these group-specific systems, many bacteria utilize the S-ribosylhomocysteine lyase (LuxS)/autoinducer-2 (AI-2) QS pathway, a widely conserved intercellular signaling mechanism that mediates both intra- and interspecies communication across G^+^ and G^−^ taxa. Figure 1 schematically summarizes these major QS communication strategies [6].

Through these conserved frameworks, QS regulates processes such as virulence expression, biofilm formation, persistence, and ecological adaptation, not only in pathogenic bacteria but also in commensal and symbiotic microbial communities, thereby contributing to cooperation, stability, and host–microbe homeostasis [6].

Recent comprehensive analyses have mapped the breadth, evolution, and mechanistic diversity of microbial QS research. In particular, the extensive review by Ruan et al. [4] provides an in-depth bibliometric and mechanistic synthesis of QS systems, detailing their molecular architectures, signaling hierarchies, and functional roles in biofilm formation, virulence regulation, symbiosis, and environmental adaptation. Building on this foundation, this section does not aim to provide an exhaustive mechanistic analysis. Instead, it offers a concise overview of the principal QS systems relevant to anti-virulence and quorum-quenching strategies.

Accordingly, the following subsections briefly summarize (i) AIP-mediated QS in G^+^ bacteria, (ii) AHL-mediated QS in G^−^ bacteria, and (iii) the LuxS/AI-2 system, which facilitates interspecies communication across both G^+^ and G^−^ bacteria. Other signaling pathways, including autoinducer-3 (AI-3)–mediated systems, are involved [29,30] in host–pathogen and inter-kingdom communication, particularly in enteric bacteria, and are acknowledged but lie beyond the scope of this review.

### 3.1. Quorum Sensing in Gram-Positive Bacteria: Autoinducing Peptide–Mediated Signaling

G^+^ bacteria predominantly employ AIPs as QS signals [31]. These short, cyclic peptides are synthesized as ribosomally encoded precursor proteins, which undergo post-translational processing and active export before engaging membrane-bound histidine kinase receptors to initiate signal transduction [31]. Because AIPs cannot freely diffuse across the cytoplasmic membrane, their maturation and secretion require specialized membrane-associated processing and transport machinery [32]. A well-characterized example is the Agr QS system of *Staphylococcus aureus* [33]. In this system, the AIP is derived from the precursor peptide AgrD through a dedicated biosynthetic pathway. The integral membrane endopeptidase AgrB cleaves the C-terminal region of AgrD and catalyzes thiolactone ring formation, while the type I signal peptidase SpsB removes the N-terminal leader sequence to release the mature, biologically active AIP into the extracellular environment [34]. Upon reaching a threshold extracellular concentration, AIPs bind to the extracellular domain of their cognate membrane-bound histidine kinase receptor, triggering receptor autophosphorylation and subsequent phosphotransfer to downstream response regulators. This phosphorylation cascade ultimately modulates transcription of QS-regulated genes controlling population-level behaviors, including biofilm formation, virulence factor expression, and stress adaptation [31].

### 3.2. Quorum Sensing in Gram-Negative Bacteria: Acyl-Homoserine Lactone–Mediated Signaling

G^−^ bacteria primarily employ small, diffusible molecules such as AHLs and other S-adenosylmethionine-derived signals that freely diffuse across membranes and bind cytoplasmic transcriptional regulators [35]. AHLs are synthesized by members of the acyl-homoserine-lactone synthase (LuxI) family of enzymes, which use two metabolic precursors: S-adenosylmethionine (SAM), which provides the homoserine lactone moiety, and an acyl group derived from acyl–ACP or, in some systems, acyl–CoA substrates [36,37]. LuxI enzymes catalyze amide bond formation between these substrates, followed by intramolecular lactonization to generate the mature AHL signal and release S-methylthioadenosine (MTA) as a by-product [38,39].

Different LuxI homologs (e.g., LasI, RhlI, AinS) exhibit substrate specificity for distinct acyl-ACPs, producing structurally diverse AHLs that vary in acyl chain length (C_4_ to C_20_), saturation, and substitution patterns (reviewed in [40]). As AHLs accumulate with increasing cell density, they reach a threshold concentration at which they bind their cognate LuxR-family transcriptional regulators [41]. LuxR proteins contain two functional domains: an N-terminal ligand-binding domain (LBD) and a C-terminal DNA-binding domain (DBD). Upon ligand binding, LuxR–AHL complexes typically become stable, dimerize, and bind target DNA sequences in a species-dependent manner to regulate transcription. Notably, for some LuxR-type regulators, autoinducer binding is required for proper folding, whereas unbound LuxR proteins may be rapidly degraded [42,43,44].

The resulting AHL–LuxR complexes form dimers or multimers that bind to specific promoter regions, thereby activating QS-regulated gene expression [45,46]. Canonical LuxI/LuxR-type systems, such as LasI/LasR and RhlI/RhlR in *P. aeruginosa*, mediate intercellular communication through endogenous AHL production and detection [47]. However, many bacteria encode LuxR-type AHL receptors even in the absence of a cognate LuxI AHL synthase. These unpaired LuxR-family proteins are termed “orphans” [48] or “solos” [49] because they function without a dedicated signal generator [42,49]. In the absence of LuxI synthases, such receptors can detect AHLs produced by other bacterial species, thereby facilitating interspecies communication [50].

Importantly, LuxI-type synthases are not essential for bacterial growth, making them attractive anti-virulence targets whose inhibition can suppress QS activation without directly affecting cell viability.

### 3.3. LuxS/AI-2–Mediated Quorum Sensing: An Interspecies Communication System in Bacteria

Beyond the species-specific systems discussed above, interspecies signaling molecules, such as AI-2, enable communication across diverse bacterial taxa. AI-2 is produced via the LuxS pathway and is conserved across G^+^ and G^−^ bacteria, mediating interspecific signaling within complex microbial communities [51]. The LuxS enzyme (EC 4.4.1.21) catalyzes the cleavage of S-ribosylhomocysteine into homocysteine and 4,5-dihydroxy-2,3-pentanedione (DPD), the precursor of AI-2, which spontaneously cyclizes into chemically distinct AI-2 derivatives depending on environmental conditions [52,53]. Importantly, AI-2 is not a single defined molecule but a family of interconverting DPD derivatives, including furanosyl borate diesters and related structures, whose chemical form depends on environmental parameters such as pH and borate availability [54]. This chemical plasticity allows AI-2 to function as a broadly conserved signaling currency rather than a species-specific ligand [55].

LuxP and LsrB are the best-characterized receptors for AI-2. Both are periplasmic substrate-binding proteins structurally related to ribose-binding proteins, despite limited primary sequence similarity. Notably, all known AI-2 receptors share high sequence similarity with, and complete conservation of the ligand-binding site with, LuxP or LsrB. [54,56]. These receptors belong to the high-affinity substrate-binding protein family but differ in their ligand specificities and downstream signaling mechanisms. Specifically, LuxP and LsrB recognize chemically distinct cyclic derivatives of the AI-2 precursor molecule DPD (S)-4,5-dihydroxypentane-2,3-dione) [54,56]. LuxP binds the borated AI-2 form, S-THMF-borate ((2S,4S)-2-methyl-2,3,3,4-tetrahydroxytetrahydrofuran-borate), whereas LsrB selectively recognizes the non-borated isomer, R-THMF ((2R,4S)-2-methyl-2,3,3,4-tetrahydroxytetrahydrofuran) [54]. This ligand discrimination is attributed to differences in the amino acid composition and architecture of the respective binding pockets.

Downstream regulatory responses also diverge according to receptor type. Upon AI-2 binding, LuxP modulates the activity of a membrane-spanning sensor histidine kinase, thereby controlling a phosphorylation-dependent signal transduction cascade. In contrast, LsrB functions as the periplasmic recognition component of an ATP-binding cassette (ABC) transporter that mediates AI-2 internalization [57,58]. LsrB was initially identified in *Salmonella enterica* serovar Typhimurium and, unlike LuxP—which appears to be restricted to members of the *Vibrionales*—is more broadly distributed among enteric bacteria and among species within the *Rhizobiaceae* and *Bacillaceae* families [59,60].

Furthermore, in *Vibrio* species, LuxP binding to AI-2 modulates the activity of the transmembrane sensor histidine kinase LuxQ, switching it from kinase to phosphatase mode and thereby regulating QS-controlled phenotypes such as bioluminescence, biofilm formation, and virulence factor production [12,61]. In contrast, in enteric bacteria such as *Escherichia coli*, the LsrB–AI-2 complex delivers AI-2 into the cytoplasm via the Lsr ATP-binding cassette transporter system, enabling intracellular signal integration [12]. LsrB-bound AI-2 can also influence chemotactic behavior through interactions with the periplasmic sensory domain of the chemoreceptor Tsr [62].

Notably, many bacteria that exhibit robust transcriptional and phenotypic responses to AI-2 lack identifiable LuxP- or LsrB-type receptors, suggesting the existence of additional, as-yet-uncharacterized AI-2 sensing mechanisms [63]. Supporting this notion, AI-2 has been shown to regulate gene expression, biofilm formation, and virulence-associated traits in pathogens such as *P. aeruginosa* and *Enterococcus faecalis*, despite the absence of canonical AI-2 receptors in these organisms [64].

## 4. Common Quorum-Sensing Mechanisms, Divergent Functional Outcomes in Pathogenic and Beneficial Bacteria

As discussed above, QS is employed not only by pathogenic bacteria but also by beneficial and symbiotic microorganisms. Although QS systems are built upon conserved molecular architectures across bacterial taxa, their biological functions and regulatory outcomes differ profoundly between pathogenic and beneficial bacteria, despite the vast diversity of bacterial lifestyles [65]. Across taxa, QS relies on the production, accumulation, detection, and interpretation of AI molecules, enabling bacterial populations to coordinate gene expression in response to cell density and community context [6]. These shared architectures encompass signal biosynthesis pathways, cognate receptors, signal transduction modules, and regulatory feedback loops that synchronize population-level behaviors. The conservation of QS machinery underscores its fundamental role in bacterial ecology rather than specialization for pathogenicity [66]. In both pathogenic and symbiotic bacteria, QS integrates environmental cues, metabolic status, and community composition to regulate collective behaviors that would be ineffective if executed by individual cells. Accordingly, QS should be regarded as a general regulatory framework for population-level coordination rather than an inherently virulence-associated system [4].

Despite this conserved signaling logic, QS circuits operate within distinct regulatory and ecological contexts that shape their biological outputs. In pathogenic bacteria, QS networks are frequently coupled to master virulence regulators and stress-response pathways, whereas in beneficial/symbiotic bacteria, they are more often embedded within metabolic, ecological, and homeostatic regulatory networks [65,67]. Consequently, identical or closely related QS architectures can govern markedly different phenotypic programs depending on bacterial lifestyle, ecological niche, evolutionary pressures, and host interactions.

This divergence between shared QS mechanisms and context-dependent regulatory outcomes is central to understanding the biological consequences of QS modulation. It explains why interference with QS signaling can strongly attenuate virulence, biofilm formation, and resistance-associated behaviors in pathogens, while eliciting more subtle, adaptive, or homeostatic effects in beneficial bacteria. These contrasts are summarized in Table 1, which provides a conceptual framework for interpreting the impact, and inherent limitations, of QS modulation, including that mediated by phytochemicals, as discussed in subsequent sections.

### 4.1. Quorum Sensing as an Amplifier of Virulence and Biofilm Formation in Pathogenic Bacteria

In pathogenic bacteria, QS functions not merely as a communication system but as a central amplifier of virulence, enabling population-wide coordination of behaviors that enhance infection, persistence, and resistance [70]. Increasing evidence indicates that bacterial communication extends beyond strict intraspecies signaling and includes interspecies “eavesdropping” and signal interception, allowing pathogens to sense and manipulate neighboring microorganisms within complex communities [71]. In pathogenic contexts, QS exploits this communicative capacity to synchronize collective behaviors that optimize infection efficiency and persistence [72].

Bacterial pathogens widely employ QS to coordinate the expression of multiple virulence-associated traits in a threshold-dependent, population-wide manner [73]. This synchronized activation ensures that virulence factors are deployed only when bacterial numbers are sufficient to overcome host defenses, thereby maximizing pathogenic success while minimizing premature immune detection. Consequently, QS circuitry in pathogenic bacteria is configured to drive abrupt and robust phenotypic transitions once critical signaling thresholds are reached.

Successful pathogenicity depends on a bacterium’s ability to adhere to and colonize host tissues, evade immune defenses, and produce toxins. QS serves as a central regulatory hub that integrates population density with environmental cues to orchestrate these energetically costly processes in a temporally and spatially controlled manner [2]. Rather than functioning as a simple on–off switch, QS integrates multiple signaling inputs to regulate toxin production, secretion-system activation, motility, immune evasion, biofilm maturation, and resistance-associated phenotypes [6].

A defining feature of QS in pathogenic bacteria is its tight coupling to virulence gene networks. In many clinically relevant species, QS systems are hierarchically organized and linked to master regulators that control extensive pathogenic regulons [74]. A well-characterized example is *P. aeruginosa*, in which AHL- and quinolone-based QS systems form interconnected regulatory circuits that govern the production of elastase, pyocyanin, rhamnolipids, lectins, and exotoxin A, as well as biofilm architecture and persistence [75,76,77,78]. Similarly, in *Staphylococcus aureus*, QS regulates the synthesis and secretion of hemolysins, protein A, enterotoxins, lipases, and fibronectin-binding proteins, all of which contribute to immune evasion and nutrient acquisition within the host [79].

Comparable QS-dependent coordination of virulence has been documented in other major pathogens, including *Vibrio* spp., *Salmonella enterica*, and *E. coli*, where QS controls toxin expression, adhesion factors, secretion systems, and motility programs essential for infection establishment and progression [73]. Collectively, these QS-regulated traits enable pathogens to overwhelm host defenses and optimize resource acquisition during infection.

QS-mediated regulation of biofilm formation represents a significant mechanism by which pathogens amplify virulence and persistence. Biofilms are structured microbial communities encased in a self-produced extracellular matrix that provides physical protection from antibiotics and immune clearance while creating microenvironments that promote stress tolerance and long-term survival [80]. QS regulates multiple stages of biofilm development, including initial surface attachment, extracellular matrix production, and biofilm maturation. Disruption of QS signaling frequently results in structurally compromised biofilms and increased susceptibility to antimicrobial agents, underscoring the central role of QS in biofilm-associated infections [81].

Beyond classical virulence traits, QS also contributes to antimicrobial resistance through several indirect but functionally significant mechanisms [72,82]. QS regulates the expression of multidrug efflux pumps, modulates stress-response pathways, and supports metabolic adaptations that enhance survival under antibiotic pressure. In addition, QS promotes horizontal gene transfer via conjugation and plasmid mobilization, thereby accelerating the dissemination of resistance determinants within pathogenic populations [83]. QS further facilitates the emergence of antibiotic persistence by enabling subsets of bacterial populations to enter transient, highly tolerant dormant states.

Taken together, these features establish QS as a virulence amplifier rather than a mere communication system in pathogenic bacteria. By synchronizing multiple virulence- and resistance-associated traits, QS enhances infection efficiency, persistence, and adaptability without directly affecting bacterial viability. This functional role provides a strong mechanistic rationale for targeting QS as an anti-virulence strategy. It sets the stage for evaluating how QS modulation, particularly by phytochemicals, may attenuate pathogenicity while avoiding the bactericidal selective pressures imposed by conventional antibiotics. This contrast becomes critical when QS functions in beneficial and symbiotic bacteria are considered in subsequent sections.

The major QS-regulated mechanisms that indirectly contribute to antimicrobial resistance in pathogenic bacteria are summarized in Table 2.

### 4.2. Quorum Sensing as a Homeostatic and Ecological Regulator in Commensal and Symbiotic Bacteria

QS systems are widely conserved among commensal, probiotic, and symbiotic members of the human microbiota, where they primarily function as regulators of cooperation, ecological fitness, and host–microbe homeostasis (reviewed in [87]). In contrast to pathogenic bacteria, where QS is often wired toward virulence amplification, QS in beneficial and symbiotic bacteria supports adaptive behaviors that promote stable colonization, metabolic coordination, and mutualistic interactions with the host. A comprehensive overview of QS systems in the intestinal microbiome and their roles in bacteria–host crosstalk has been provided by Wu and Luo [68], who summarize current evidence linking QS signaling to the maintenance of intestinal homeostasis under physiological conditions. Building on this foundation, the present section focuses on the broader ecological and homeostatic functions of QS in commensal and symbiotic bacteria, with particular emphasis on the implications of QS-targeted interventions.

Within the gastrointestinal tract, QS plays a key role in shaping the spatial organization and ecological structure of microbial populations. The gut represents a highly heterogeneous environment in which microbial density, nutrient availability, and physical architecture vary along its length and across distinct niches, including the mucus layer and the intestinal lumen. Experimental and modeling studies indicate that microbial aggregate size and population density influence both the quorum threshold required for effective QS signaling and the spatial range over which QS signals affect neighboring species [88,89]. In this context, QS enables bacteria to sense local population structure and dynamically adapt their behavior to specific ecological niches.

Among QS pathways, the LuxS/AI-2 system has emerged as a central mediator of interspecies communication within the gut microbiota. LuxS-dependent AI-2 production is critical for gastrointestinal transit, adhesion, and biofilm formation in *Lactobacillus rhamnosus* GG, with *luxS* deletion resulting in impaired colonization and reduced biofilm development [90,91]. AI-2 signaling further influences mucosal adhesion, biofilm formation, and iron metabolism in beneficial genera such as *Lactobacillus* and *Bifidobacterium*, as well as in *Actinobacillus* and *Vibrio* species [92,93,94]. Collectively, these findings suggest that AI-2 contributes to niche adaptation and efficient resource utilization within complex microbial communities. In LAB, LuxS/AI-2 signaling supports probiotic traits, including colonization persistence and cooperative behavior. For example, AI-2 enhances bacteriocin production in co-cultures of *Lactobacillus plantarum* AB-1 and *Lactobacillus casei* [95], promotes intestinal adhesion of *L. plantarum* KLDS 1.0391, and supports biofilm formation in *L. rhamnosus* GG [96].

QS also contributes to microbiota stability by modulating interactions with the host immune system. Gut microbes exert profound effects on host metabolic and immune functions, and QS-mediated signaling has been implicated in cross-kingdom communication between bacteria and intestinal epithelial or immune cells [68,97]. In healthy individuals, balanced QS signaling supports host–microbe homeostasis, whereas dysbiosis is associated with altered QS signal profiles. Notably, the abundance and composition of AHLs in fecal samples differ significantly between healthy individuals and patients with inflammatory bowel disease (IBD) [68]. One specific AHL, 3-oxo-C12:2-HSL, is markedly reduced in IBD patients and exhibits anti-inflammatory effects in intestinal epithelial cell models, suggesting that QS signals may serve as biomarkers of gut homeostasis and inflammatory status [98].

In addition to bacterial-derived signals, host cells participate in QS-like communication in the gut by producing AI-2–mimicking molecules in response to epithelial damage [99]. These host-derived cues serve as ecological signals sensed by diverse gut microbes, including commensals and opportunistic pathogens. Because AI-2 is a broadly conserved communication molecule, such mimics activate bacterial QS pathways across taxa. In enteric bacteria such as *S. typhimurium*, these signals are detected by the Lsr system, which triggers QS-regulated gene expression programs associated with metabolism, motility, and stress adaptation, rather than directly activating virulence. Although the precise ecological consequences of this signaling remain incompletely defined, host-driven QS activation may facilitate microbial organization and colonization at damaged epithelial sites, thereby supporting tissue repair and barrier recovery. In this context, pathogen responsiveness likely reflects signal exploitation rather than host intent. Collectively, these observations highlight the bidirectional nature of QS-mediated host–microbe interactions and their role in maintaining intestinal stability and homeostasis [99].

QS-regulated homeostatic functions extend beyond the gut to other mucosal environments, including the vaginal tract. Dominant vaginal *Lactobacillus* species, such as *L. crispatus*, *L. gasseri*, *L. iners*, and *L. jensenii*, form biofilm networks that protect against pathogen colonization and maintain low vaginal pH through lactic acid production [100,101]. Emerging evidence suggests that QS-regulated biofilm formation is critical for vaginal health. QS activity has been linked to epithelial adhesion and biofilm formation in *Lactobacillus* species, reinforcing mucosal barrier integrity [102]. Notably, recent studies have detected AHL production in certain G^+^ vaginal lactobacilli, including *L. crispatus* and *L. jensenii*, with higher AHL levels correlating with enhanced biofilm formation. Although preliminary, these findings suggest that non-canonical QS signaling may contribute to probiotic fitness in the vaginal niche [103].

Collectively, the evidence above supports a central translational principle: QS in commensal and symbiotic bacteria is primarily embedded in networks that stabilize colonization, resource sharing, and host–microbe homeostasis, so indiscriminate QS attenuation may carry ecological costs even when virulence is not the target. This implies that the therapeutic value of QS inhibition will depend not only on anti-virulence efficacy in pathogens but also on context-dependent selectivity within complex microbiome ecosystems. At present, this risk–benefit balance remains difficult to predict because most QSI studies rely on simplified pathogen-centric models and rarely quantify impacts on commensal fitness, community structure, or host signaling. Integrating microbiome-aware readouts and physiologically relevant models into QSI evaluation will therefore be essential to ensure that QS-targeted strategies, particularly phytochemical modulators, attenuate pathogenic behaviors without eroding beneficial microbial functions.

Key QS mechanisms involved in colonization, metabolism, immune modulation, and stability are summarized in Table 3.

## 5. Phytochemical Modulation of Quorum Sensing in Pathogenic Bacteria: Mechanistic Insights and Limitations

Phytochemical modulation of QS has been widely investigated as an anti-virulence strategy to suppress pathogenic behaviors, particularly the production of virulence factors and biofilm maturation. The disruption of QS, broadly termed QQ, encompasses any process that interferes with bacterial communication [105]. Mechanisms generally fall into two categories: enzymatic degradation of signaling molecules (often referred to as QQ enzymes) or small-molecule QSIs that perturb signaling without degrading the signal itself [106]. Interventions are typically classified by their primary functional target: (i) the synthesis or stability of AI molecules, (ii) signal perception at QS receptors, or (iii) downstream regulatory pathways [107,108]. This section focuses primarily on phytochemical interference with AI biosynthesis/availability and receptor engagement. These represent the most proximal and QS-specific intervention points, whereas downstream modulation is addressed only briefly, as it often converges on broader, less specific metabolic networks [109].

A central question arising from the conserved nature of QS systems discussed in Section 3 and Section 4 is: Can phytochemicals modulate QS with sufficient mechanistic specificity to attenuate pathogen behaviors while minimizing unintended ecological effects? Although diverse compound classes, such as phenolics, have demonstrated the capacity to inhibit QS-regulated behaviors across multiple strains [6,22,69,109], distinguishing specific mechanistic interference from general toxicity remains a challenge. Consequently, the following subsections synthesize pathogen-focused mechanistic evidence while critically evaluating the experimental constraints, such as reliance on indirect phenotypic endpoints and limited molecular target validation, that currently hinder the translation of these findings from proof-of-concept studies to viable anti-virulence strategies [110,111,112,113].

### 5.1. Phytochemical Inhibition of Autoinducer Biosynthetic Machinery

Disrupting QS by inhibiting AI biosynthesis, thereby targeting the molecular machinery responsible for signal production rather than the signaling molecules themselves, represents a promising anti-virulence strategy. However, the feasibility and mechanistic basis of this approach differ substantially across QS systems. Accordingly, the following subsections examine the modulation of AI biosynthesis by phytochemicals in G^+^-AIP systems, G^−^-AHL systems, and the universally conserved LuxS/AI-2 pathway.

#### 5.1.1. Phytochemical Modulation of Autoinducing Peptide Biosynthesis in Gram-Positive Bacteria

In G^+^ bacteria, inhibiting AIPs biosynthesis poses substantial mechanistic and translational challenges. AIPs are ribosomally synthesized precursor peptides that undergo post-translational processing by dedicated enzymes (e.g., AgrD/AgrB in staphylococci). These processing steps are essential for AIP production but not generally required for bacterial viability. By contrast, inhibition of broadly essential secretion or signal-peptidase enzymes can be bactericidal because those enzymes process many substrates beyond AIPs. Therefore, direct inhibition of AIP-specific biosynthetic components is typically an anti-virulence approach rather than a bactericidal one, and the therapeutic and evolutionary consequences differ accordingly [114]. This constraint has contributed to the relative paucity of studies directly targeting AIP biosynthesis in G^+^ bacteria.

Despite these limitations, considerable effort has been devoted to elucidating the molecular mechanisms governing AIP production, processing, and signal transduction, to identify regulatory nodes that can be modulated without directly compromising bacterial viability [115]. In this context, indirect interference with AIP biosynthesis or the agr system, rather than direct inhibition of ribosomal activity or essential peptidases, has emerged as a more feasible anti-virulence strategy.

Notably, phytochemicals have been reported to modulate AIP-dependent QS systems through indirect mechanisms. For example, polyphenolic compounds including curcumin and resveratrol have been shown to downregulate AIP biosynthesis and impair agr-mediated QS signaling in *S. aureus*, leading to reduced expression of QS-regulated virulence factors without overt effects on bacterial growth [116]. Collectively, these findings indicate that although direct inhibition of AIP biosynthesis remains problematic from a translational perspective, selective modulation of QS peptide signaling through upstream regulatory interference or signal attenuation may be a viable anti-virulence strategy. Importantly, these examples underscore the need for careful mechanistic dissection to distinguish genuine QS modulation from secondary effects arising from growth inhibition or metabolic stress.

Importantly, in contrast to AIP-based systems, certain QSIs directly target autoinducer synthases in LuxI/AHL-dependent systems of G^−^ bacteria [117], as well as the LuxS enzyme in the ‘universal’ AI-2–dependent system of both G^−^ and G^+^ bacteria, thereby preventing signal biosynthesis and subsequent QS activation [118]. These mechanisms are discussed in detail in the following subsections.

#### 5.1.2. Phytochemical Modulation of Acyl-Homoserine Lactone Biosynthesis in Gram-Negative Bacteria

The AHL–mediated QS system is the most extensively characterized QS mechanism in G^−^ bacteria and represents a central regulatory pathway in many clinically relevant pathogens. In these organisms, QS signaling is primarily mediated by AHLs, which accumulate extracellularly in a cell-density-dependent manner. Upon reaching a threshold concentration, AHLs diffuse back into the cell and bind to the cytoplasmic LuxR-type transcriptional regulators, which activate QS-controlled gene expression programs that govern virulence, biofilm formation, and stress adaptation [12].

A growing body of evidence demonstrates that diverse phytochemicals can interfere with AHL biosynthesis by targeting LuxI-type enzymes or their transcriptional regulation. Inhibition of AHL synthase activity blocks QS signaling upstream of receptor activation, thereby preventing induction of QS-regulated genes and phenotypes [119]. Representative phytochemicals reported to modulate LuxI-dependent AHL biosynthesis in G^−^ bacteria are summarized in Table 4.

Among terpenoid compounds, carvacrol has been extensively investigated as a QS inhibitor. Burt et al. [120] demonstrated that sub-MICs of carvacrol suppress expression of *cviI*, the LuxI homolog in *Chromobacterium violaceum*, leading to reduced AHL production and downregulation of QS-dependent outputs, such as violacein synthesis and chitinase activity. Complementary findings in *P. aeruginosa* further indicate that carvacrol attenuates QS-regulated virulence phenotypes by disrupting AHL-dependent signaling pathways, consistent with indirect interference with LuxI-mediated autoinducer synthesis [121].

Structurally related monoterpenes exhibit similar effects. For example, Li et al. [122] showed that L-carvone significantly reduced AHL production in *Hafnia alvei* by downregulating both the AHL synthase gene *halI* and its cognate regulator *halR*, leading to impaired motility and biofilm formation. These findings underscore the capacity of small terpenoids to modulate QS at the level of signal biosynthesis and regulatory feedback.

Phenylpropanoid derivatives also display LuxI-targeting activity. Salicylic acid suppresses AHL production in *P. aeruginosa* and interferes with both the Las and Rhl QS systems, thereby reducing bacterial invasion and cytotoxicity in epithelial cell models [123,124]. Similarly, trans-cinnamaldehyde downregulates *lasI*, *lasR*, *rhlI*, and *rhlR* expression in *P. aeruginosa* PAO1 at sub-inhibitory concentrations, leading to substantial reductions in protease, elastase, pyocyanin, rhamnolipid production, and biofilm formation without bactericidal effects [125].

**Table 4:** Examples of phytochemicals reported to suppress AHL (LuxI-type) autoinducer biosynthesis in Gram-negative bacteria.

Phytochemical (Class)	Model Organism/ QS System	Putative Mechanism (AHL Biosynthesis-Related)	Key Outcomes	Refs.
Carvacrol (terpenoid)	*Chromobacterium violaceum* (CviI/CviR; AHL QS)	Downregulates AHL synthase gene (cviI) expression at sub-MIC Decreases AHL accumulation upstream of receptor activation (transcriptional/production-level interference).	Reduced AHL-dependent violacein and chitinase; inhibited biofilm at sub-MIC.	[120]
L-carvone (terpenoid)	*Hafnia alvei* (HalI/HalR; AHL QS)	Suppresses AHL production by downregulating halI (AHL synthase) and halR (regulator) Upstream inhibition of QS activation	Reduced motility and biofilm formation; decreased AHL signal output.	[122]
Salicylic acid (Phenolic acid)	*P. aeruginosa* (Las/Rhl systems) and ocular infection model	Represses AHL production and QS gene expression (lasI/rhlI/lasR/rhlR) at sub-inhibitory level Reduces AHL output in whole-cell assays and in heterologous LasI/RhlI production systems, as shown by analytical AHL quantification (LC–MS) in some studies	Reduced AHL output and QS-regulated virulence traits; reduced host–cell invasion/cytotoxicity in epithelial models.	[123,125,126,127]
trans-Cinnamaldehyde (Phenylpropanoid)	*P. aeruginosa* (LasI/LasR and RhlI/RhlR)	Downregulates lasI/rhlI and associated QS regulatory genes at sub-inhibitory concentrations Reduces AHL signal output in whole-cell systems and support from heterologous LasI/RhlI assays with AHL quantification (LC–MS) and docking in some studies	Reduced protease, elastase, pyocyanin; reduced biofilm; reduced QS gene expression.	[125,127]
Tannic acid (polyphenol)	*P. aeruginosa* (Las/Rhl systems); heterologous LasI/RhlI AHL-production assay (reported)	Reduced AHL accumulation in LasI/RhlI-dependent assays (notably abolishing short-chain RhlI AHLs), consistent with interference in AHL biosynthesis Direct LuxI enzymatic inhibition was not demonstrated (no purified-enzyme kinetics)	Reduced AHL signal output (biosensor/analytical) and QS-regulated phenotypes (reported).	[127]
Eugenol (phenylpropanoid)	*P. aeruginosa* (LasI/RhlI; AHL QS)	Reduces 3-oxo-C12-HSL and C4-HSL levels and downregulates *lasI/rhlI* at sub-MIC Consistent with upstream suppression of AHL biosynthesis.	Decreased pyocyanin, swarming, and rhamnolipid production; inhibited biofilm (nanoemulsion higher effect than free eugenol)	[128]

In addition to simple phenylpropanoids, higher-order polyphenols have also been shown to interfere with AHL-dependent quorum sensing. Chang et al. [127] demonstrated growth-neutral inhibition of short-chain AHL production by tannic acid in their screening platform; however, the authors did not include *luxI/lasI/rhlI* expression or protein measurements, nor biofilm assays, so the mechanism and effects on QS-regulated biofilm remain to be established.

Eugenol provides another illustrative example of phytochemical-mediated inhibition of AHL biosynthesis. Lou et al. [128] demonstrated that eugenol and an optimized eugenol nanoemulsion significantly reduced AHL levels (3-oxo-C12-HSL and C4-HSL) in *P. aeruginosa* by downregulating *lasI* and *rhlI* expression, thereby suppressing QS-regulated virulence factors, swarming motility, and biofilm formation. Notably, nanoemulsion delivery enhanced eugenol’s anti-QS efficacy without affecting bacterial growth, highlighting formulation-dependent effects on QS.

Collectively, these studies demonstrate that inhibition of LuxI-dependent AHL biosynthesis is a mechanistically plausible and experimentally supported strategy for QS modulation in G^−^ pathogens. However, important limitations remain. Reported effects are frequently strain-specific and highly dependent on experimental conditions, including growth phase, assay system, and compound formulation. Moreover, relatively few studies directly interrogate LuxI enzymatic activity or confirm target engagement at the molecular level, compared with the large body of literature documenting phenotypic suppression of QS-regulated outputs.

This disparity raises a critical unresolved question: whether phytochemicals directly inhibit QS machinery or exert indirect effects through broader metabolic or stress-related pathways. Given the pleiotropic nature of many phenolic and terpenoid compounds, definitive attribution of QS inhibition to LuxI-targeted mechanisms requires rigorous biochemical validation. Addressing these methodological gaps will be essential for advancing phytochemical QS inhibitors toward translational anti-virulence applications.

Finally, although LuxI-type AHL synthases are mostly associated with G^−^ pathogens, they are also found in a subset of non-pathogenic and symbiotic G^−^ bacteria, where AHL signaling typically regulates colonization, metabolic coordination, or environmental adaptation rather than virulence. In contrast, many beneficial bacteria, particularly G^+^ probiotics, lack LuxI homologs and instead rely on peptide-based or LuxS/AI-2 quorum-sensing systems. These distinctions have important implications for the microbiome-level consequences of LuxI-targeted QS inhibition, as considered in subsequent sections.

#### 5.1.3. Phytochemical Inhibition of LuxS-Dependent AI-2 Biosynthesis: A Conserved Target Across Gram-Positive and Gram-Negative Bacteria

A unique, evolutionarily conserved QS mechanism shared by both G^+^ and G^−^ bacteria is the production of AI-2 signaling molecules by the enzyme LuxS. Because AI-2 mediates interspecies communication, the LuxS/AI-2 system occupies a central role within complex microbial communities. Moreover, the LuxS/AI-2 system is widely distributed across phylogeny and is present in diverse bacterial taxa, including *E. coli*, *S. aureus*, *Helicobacter pylori*, *Bacillus subtilis*, and numerous commensal bacteria [51]. At the molecular level, LuxS is a metalloenzyme with a highly conserved metal-binding catalytic pocket, a feature that underpins both its enzymatic efficiency and its attractiveness as a pharmacological target [51,129]. Importantly, LuxS-mediated signaling regulates not only virulence and biofilm formation but also motility, stress responses, and metabolic coordination, depending on the bacterial lifestyle and ecological context [63,130].

Given these features, LuxS represents a promising anti-virulence target, particularly because its inhibition attenuates QS-regulated behaviors without directly impairing bacterial viability. This conceptual advantage has driven growing interest in phytochemicals as potential LuxS inhibitors, offering an alternative strategy to combat multidrug-resistant pathogens while minimizing selective pressure for resistance development.

Early evidence supporting AI-2–focused QS inhibition emerged from studies using plant extracts. For example, grape seed extract was shown to suppress AI-2–dependent behaviors in *E. coli*, including flagellar motility and Shiga toxin production [131], while extracts from broccoli, oregano, rosemary, turmeric, ginger, and basil inhibited swarming motility by disrupting the AI-2 signaling axis [109,132]. These findings established that AI-2 signalling can be modulated by complex phytochemical mixtures.

Building on this foundation, our group evaluated the anti-QS activity of ethanolic extracts from eight aromatic plants in the Cypriot flora, focusing specifically on AI-2–mediated QS in *E. coli* MG1655 [133]. Extracts from oregano, rosemary, and common sage were the most potent, suppressing AI-2 signaling by 60–90% in a dose-dependent manner. These effects were accompanied by marked reductions in biofilm formation and swimming/swarming motility, while bacterial growth remained unaffected—indicating genuine QS inhibition rather than bacteriostatic activity. LC-MS profiling identified shared phytochemicals, including carnosol, chlorogenic acid, and quercetin, suggesting these compounds as key contributors to LuxS modulation.

A subsequent mechanistic study from our group [118] extended these observations by interrogating purified phytochemicals using an integrated in silico–in vitro pipeline. Our molecular docking analysis revealed stable binding of carnosol, chlorogenic acid, quercetin, apigenin, and rosmarinic acid within the LuxS catalytic pocket. These predictions were experimentally validated with targeted biochemical assays, which confirmed reduced AI-2 production and impaired biofilm formation without growth inhibition—providing direct evidence of LuxS-targeted anti-QS activity. Notably, carnosol and chlorogenic acid showed the most potent effects, with IC_50_ values of approximately 60 μM.

Independent studies further support the general applicability of this mechanism. Fernandes et al. [134] identified curcumin and 10-undecenoic acid as inhibitors of LuxS/AI-2 signaling in *B. subtilis*, reporting concentration-dependent reductions in AI-2 activity ranging from 33–77% for curcumin and 36–64% for 10-undecenoic acid. Notably, both compounds also inhibited the LasI/LasR system in *P. aeruginosa*, highlighting their capacity to target multiple QS pathways across G^+^ and G^−^ bacteria.

Morgan et al. [132] demonstrated that carvacrol, cinnamaldehyde, and eugenol suppress QS-regulated motility and biofilm formation in multidrug-resistant uropathogenic *E. coli* (UPEC), concomitant with downregulation of luxS expression. Importantly, combining these phytochemicals with conventional antibiotics reduced antibiotic MICs and produced synergistic or partially synergistic effects, suggesting translational potential as QS-sensitizing adjuvants.

More recently, computational and molecular dynamics studies by Rath et al. [135] explored Triphala, a polyherbal formulation, as a LuxS-targeting agent in *H. pylori*. Several constituent phytochemicals—particularly furosin, corilagin, and ellagic acid—exhibited higher predicted LuxS binding affinity and stability than the reference antibiotic clarithromycin, with furosin emerging as the most stable inhibitor. These findings bridge traditional herbal medicine with modern structure-guided QS inhibitor discovery and further validate LuxS as a druggable QS target.

Representative studies targeting LuxS-dependent AI-2 biosynthesis are summarized in Table 5.

Current evidence shows LuxS-dependent AI-2 biosynthesis as a promising and conserved target for phytochemical-based QS inhibition. Unlike peptide- or AHL-based systems, LuxS is found in both pathogenic and non-pathogenic bacteria, linking AI-2 modulation to virulence and microbiome regulation. Many phytochemicals, such as polyphenols, terpenoids, and herbal formulations, have shown LuxS-linked anti-QS activity, but rigorous validation is limited. Differentiating direct LuxS inhibition from indirect effects is crucial for progress. LuxS remains a rare yet promising target for microbiome-aware anti-virulence strategies, particularly as future research integrates enzymology, structural biology, and in vivo studies.

### 5.2. Targeting Quorum-Sensing Receptors with Phytochemicals

The second mechanism of QS-inhibiting agents is to target QS signaling receptors, thereby inactivating them or competing for them. In most cases, the ligand-binding domains of receptors with the native AIs are highly conserved and can be bound by most AIs either competitively or noncompetitively [136].

#### 5.2.1. Phytochemical Antagonists and Allosteric Modulators of AIP Receptors in Gram-Positive Bacteria

G^+^ bacteria typically detect secreted cyclic peptide AIPs via transmembrane sensor kinases embedded in two-component regulatory systems (for example, AgrC in *Staphylococcus* spp.), which transduce QS signals into gene-expression programs governing community behaviors such as virulence and biofilm formation [137]. These receptors can discriminate subtle sequence differences among AIPs, supporting largely species-specific communication; indeed, AIP-mediated QS systems (for example, the four thiolactone peptides AIP I–IV in *S. aureus*) underlie this specificity. In *S. aureus*, the accessory gene regulator (agr) pathway synthesizes the cyclic thiolactone pheromone from the AgrD precursor, exports it via AgrB, and detects it through the AgrC/AgrA two-component system. Upon AIP binding, AgrC activates the response regulator AgrA, which binds the agr promoters (P2 for RNAII and P3 for RNAIII) to coordinate downstream transcriptional outputs [138]. Consequently, QS-mediated pathogenicity in G^+^ bacteria can be attenuated by targeting AIP receptors with antagonists, and emerging evidence supports inhibition of the AgrC–AIP interaction as a promising therapeutic strategy [19,139]. Small-molecule kinase inhibitors, including closantel, RWJ-49815, and LY266500, have also been shown to disrupt QS signaling in G^+^ bacteria by interfering with receptor kinase activity [140].

Although multiple phytochemicals have been reported to suppress AIP-dependent agr outputs (e.g., biofilm formation, RNAIII, phenol-soluble modulins), direct evidence that plant-derived small molecules competitively bind or allosterically modulate AgrC remains limited. Most studies describe transcriptional or phenotypic inhibition without supporting biochemical binding assays, structural validation, or receptor mutagenesis. For example, Nakagawa et al. [141] reported that carnosic acid and carnosol from *Rosmarinus officinalis* (rosemary) specifically inhibit AIP-induced agr RNAIII and psmα expression in *S. aureus* reporter strains and in clinical isolates from patients with atopic dermatitis. Using luciferase reporter assays and qPCR, inhibition was observed at low micromolar concentrations for the purified compounds and at low µg/mL for rosemary extracts, with minimal impact on bacterial growth at the tested doses. The authors evaluated nine commercial rosemary extracts) and observed that activity correlated with carnosic acid/carnosol content; they further discussed topical anti-virulence applications in atopic dermatitis, while explicitly noting the absence of direct biochemical evidence for AgrC receptor binding. Given the limited number of studies and the lack of mechanistic validation at the receptor level, potential AIP receptor inhibitors are not discussed further herein.

#### 5.2.2. Phytochemical Antagonism and Allosteric Modulation of LuxR-Family AHL Receptors in Gram-Negative Pathogens

In G^−^ bacteria (particularly Proteobacteria), the most common AHL receptors are LuxR-family transcription factors that bind AHLs to form LuxR–AHL complexes that regulate gene expression. Membrane sensor kinases and LuxR-solo (orphan) variants also contribute to AHL/autoinducer sensing in many species [50,142]. Targeting LuxR–AHL recognition with specific QSIs, therefore, represents a promising anti-virulence strategy. Notably, because LuxR homologs vary in ligand specificity and some bacteria employ alternative receptor architectures, combining receptor-targeting QSIs with strategies that inhibit signal synthesis or promote signal degradation can broaden efficacy and reduce the risk of escape.

Accordingly, disrupting LuxR–AHL recognition is a prominent anti-virulence strategy. Because LuxR homologs differ in ligand specificity and some pathogens employ alternative receptor architectures, receptor-directed QSIs are often best considered alongside complementary approaches that affect signal availability (e.g., synthesis inhibition or signal quenching), which may broaden efficacy and reduce the likelihood of escape [19,143]. As an illustrative example, the LasR–AHL system in *P. aeruginosa* is a central regulator of virulence and biofilm formation; LasR inhibition can attenuate AHL-dependent transcriptional programs and impair biofilm-associated phenotypes in this pathogen [144]. Table 6 summarizes representative compounds reported to interfere with LasR-mediated signaling in *P. aeruginosa* and related G^−^ bacteria and highlights the range of supporting evidence, from in silico prioritization to biochemical receptor assays and phenotypic validation.

A growing subset of reports uses quantitative structure-activity relationship (QSAR)/virtual screening, docking, and molecular dynamics to nominate phytochemical LasR antagonists. For example, Ali et al. [145] screened a library of 2727 phytochemicals against the LasR AHL receptor of *P. aeruginosa* using a QSAR-guided virtual screening pipeline combined with molecular docking and molecular dynamics. They identified several candidates with stronger LasR-binding profiles than the reference inhibitor FY1, particularly compounds 5,281,647, 57,331,045, and 52,81,672, which showed strong docking affinities, stable hydrogen-bonding networks, and favorable free-energy landscapes. Molecular dynamics simulations (200 ns) supported stable ligand–LasR complexes with sustained interactions involving key residues (e.g., Trp60, Asp73, Thr115, Ser129), underscoring the potential of phytochemicals as LasR-targeting QSIs that can interfere with AHL-mediated receptor activation and virulence regulation.

Almatroudi et al. [146] used a machine-learning–guided virtual screening strategy with docking, molecular dynamics, and MM/PBSA analyses to identify LasR inhibitors from a library of 9000 phytochemicals. A Random Forest classifier predicted 367 potential actives, of which 155 met drug-likeness criteria. Docking highlighted three top candidates—PubChem 3,795,981, 42,607,867, and 697,066—with strong predicted binding affinities (−11.8 to −12.0 kcal/mol) and consistent hydrogen bonding to residues important for pocket stabilization (e.g., Tyr56, Trp60, Thr75, Thr115, Ser129). Subsequent simulations indicated that PubChem 3,795,981 and 42,607,867 maintained particularly stable interactions and favorable binding free energies, supporting their potential as phytochemical LasR antagonists capable of disrupting AHL-dependent signaling and biofilm-associated virulence.

These computational studies are valuable for hypothesis generation and scaffold prioritization, but predicted binding affinity alone does not establish biological QS inhibition. Docking outputs do not capture key determinants of bacterial activity, including permeability barriers, efflux, compound stability in complex media, and growth-phase–dependent receptor availability; therefore, they require orthogonal experimental verification before target engagement can be inferred. In addition, series-level consistency (i.e., coherent structure–activity trends across related analogs) is generally more informative than single-compound docking scores when prioritizing candidates for development.

Experimental work provides stronger support for receptor-level mechanisms in several cases. The inhibitory activity of flavonoids against the autoinducer-binding receptors LasR and RhlR has been biochemically validated by Paczkowski et al. [147]. Nine flavonoids (phloretin, chrysin, naringenin, quercetin, baicalein, apigenin, 7,8-dihydroxyflavone, 3,5,7-trihydroxyflavone, and pinocembrin) were shown to inhibit QS via an allosteric, non-competitive mechanism, binding to sites on LasR/RhlR distinct from the canonical autoinducer pocket and thereby impairing receptor function. Structure–activity relationship analyses further indicated that two hydroxyl moieties in the flavone A-ring are critical for potent inhibition, providing a practical criterion for screening future phenolic compounds.

Kim et al. [148] applied an in silico approach, predicting that gingerol can bind the LasR QS regulator. Using standard phenotypic assays, they subsequently observed reduced production of multiple virulence factors and decreased biofilm formation after gingerol exposure, consistent with interference with the binding of the cognate signal molecule, 3-oxo-C12-HSL, to LasR.

Mechanistic complexity is further supported by Hernando-Amado et al. [149], who reported that naringenin inhibits the *P. aeruginosa* QS response through time-dependent competition with 3-oxo-C12-HSL. When added at time zero, naringenin inhibited QS-regulated genes and virulence factors by binding nascent LasR. In contrast, when introduced at the stationary phase, QS outputs were not inhibited because LasR had already been activated by 3-oxo-C12-HSL. These findings emphasize the importance of identifying QSIs that remain effective in dense, late-stage populations, rather than solely suppressing early phenotypes.

Quecan et al. [150] examined quercetin-rich onion extracts and major phenolics (quercetin aglycone and quercetin 3-β-D-glucoside) in *Chromobacterium violaceum*, *P. aeruginosa*, and *Serratia marcescens*. Red onion extract and quercetin aglycone significantly inhibited violacein production in *C. violaceum*, while both quercetin forms reduced swarming motility in *P. aeruginosa* and *S. marcescens*. Docking suggested that quercetin aglycone fits more effectively into the CviR receptor pocket than the glycosylated form, whereas both compounds could bind LasR. However, neither the extracts nor isolated quercetins inhibited biofilm formation at sub-MIC levels.

Collectively, these studies demonstrate that phytochemicals, particularly flavonoids and related phenolic compounds, can modulate QS pathways in *P. aeruginosa* through diverse receptor-level mechanisms, including competitive, non-competitive, and time-dependent inhibition of LuxR-type regulators. Experimental validation alongside computational screening highlights LasR as a recurrent and tractable molecular target, while structure–activity relationships provide actionable criteria for prioritizing candidate scaffolds. At the same time, variability in phenotypic outcomes, dependence on growth phase, and limited efficacy against mature biofilms underscore the mechanistic complexity of QS inhibition and caution against assuming uniform anti-virulence effects. Future efforts integrating high-resolution receptor assays, temporal dynamics, and in vivo validation will be essential to distinguish broadly effective QS inhibitors from context-dependent modulators and to advance phytochemical QSIs toward translational relevance.

#### 5.2.3. Targeting AI-2 Receptors: Opportunities and Current Limitations

In addition to inhibiting AI-2 biosynthesis via LuxS, an alternative strategy for modulating AI-2–mediated QS involves targeting AI-2 signal perception at the receptor level. AI-2 detection is mediated by distinct periplasmic receptor systems across bacterial taxa, including LuxP in *Vibrio harveyi*, LsrB in *S. typhimurium* and *E. coli*, and the ribose-binding protein RbsB in *Aggregatibacter actinomycetemcomitans* [151]. These receptors initiate downstream QS responses that regulate bioluminescence, virulence, motility, and biofilm formation, making them conceptually attractive targets for QS interference.

Proof-of-concept studies using synthetic or non-phytochemical compounds have demonstrated that AI-2 receptor antagonism is feasible. For example, a sulfone-based molecule has been reported to function as an antagonist of the LuxP receptor in *V. harveyi*, while aromatic compounds, such as polyols and phenylboronic derivatives, reduce AI-2–dependent bioluminescence at subinhibitory concentrations. Similarly, the nucleoside analogue LMC-21 suppresses QS-regulated pigment and protease production in *Vibrio anguillarum* and inhibits biofilm formation in *V. cholerae*, *V. vulnificus*, and *V. anguillarum* [19]. In periodontal pathogens, D-galactose has been shown to reduce AI-2 activity and biofilm formation, consistent with interference at the level of AI-2 recognition. This effect is mechanistically plausible, as the AI-2 receptor RbsB shares structural similarity with D-galactose-binding proteins [152].

More recently, peptide-based approaches have been explored as alternatives to disrupt AI-2 signaling. A short peptide (designated 5906) was identified that binds LuxS and interferes with LuxS dimerization, thereby indirectly suppressing AI-2 production and QS signaling in *Edwardsiella tarda* [51]. While this strategy targets the enzyme rather than the receptor per se, it underscores the conceptual appeal of disrupting protein–protein interactions within the AI-2 signaling axis.

Despite these advances, receptor-level targeting of AI-2 signaling presents substantial conceptual and experimental challenges. LuxP functions as a periplasmic AI-2 receptor in *V. harveyi* and is a central component of a canonical two-component signaling system [56]. However, *luxP* deletion mutants retain partial AI-2 responsiveness through alternative receptor systems and parallel metabolic inputs, highlighting the redundancy and robustness of AI-2 sensing networks. In addition to LuxP, AI-2 uptake and sensing can be mediated by LsrB in *E. coli*, *Salmonella enterica*, and *Bacillus* species, and by RbsB-like proteins in other taxa. Furthermore, genomic analyses indicate that LuxP-like homologs are present in several G^+^ bacteria, although their functional roles and ecological relevance remain poorly defined [63,153].

This receptor heterogeneity complicates the development of broadly effective AI-2 receptor antagonists. In *Vibrio* species, multiple Lux homologs contribute to QS regulation, yet the precise division of labor among these components—particularly in species such as *V. parahaemolyticus*—remains incompletely resolved [154]. As a result, it is difficult to predict whether inhibition of a single AI-2 receptor would produce robust or durable QS suppression across strains or ecological contexts.

Importantly, there is currently no convincing experimental evidence demonstrating direct binding or functional antagonism of LuxP or LsrB by phytochemicals. While numerous plant-derived compounds suppress AI-2–dependent QS phenotypes, these effects are almost invariably attributed to inhibition of LuxS activity, interference with upstream metabolism, or downstream modulation of QS-regulated gene expression. Direct receptor-level inhibition by phytochemicals remains largely unexplored and represents a significant knowledge gap.

Targeting AI-2 receptors represents a theoretically attractive but experimentally underdeveloped strategy for QS modulation. While synthetic molecules and carbohydrate analogues demonstrate that receptor antagonism is possible, phytochemical-mediated inhibition of AI-2 receptors has not yet been convincingly demonstrated. The structural diversity and redundancy of AI-2 receptor systems, coupled with incomplete functional annotation across bacterial taxa, pose major barriers to translation. Consequently, current evidence supports LuxS inhibition as the primary and most substantiated phytochemical entry point into AI-2 quorum sensing, whereas receptor-level targeting remains a speculative but potentially valuable avenue for future research.

#### 5.2.4. In Silico Insights: Structural Determinants and Methodological Limitations

Computational docking and QSAR analyses are increasingly used to prioritize phytochemical QSIs, enabling high-throughput triage of large natural product libraries. Recent modeling studies discussed above [145,146] suggest convergent structural features associated with putative QS inhibition, particularly for LuxR-type receptors. A recurring observation is the importance of hydrogen-bonding networks within the ligand-binding domain (LBD): virtual screening campaigns against *P. aeruginosa* LasR frequently identify interactions with conserved LBD residues as key contributors to predicted affinity and pose stability [145,146]. Many candidate scaffolds are also predicted to exploit the hydrophobic character of the AHL-binding pocket, in some cases partially mimicking the lactone/core geometry and hydrophobic substituent pattern of native AHL ligands, thereby generating testable hypotheses regarding pharmacophore requirements for competitive or destabilizing receptor modulation [155].

However, the reliability of docking-based inference varies markedly by target class. Soluble LuxR-family LBDs (e.g., LasR, TraR) are comparatively tractable because structural templates support more informative pose predictions [156]. In contrast, membrane-embedded sensors, such as histidine kinases (e.g., AgrC), introduce substantial uncertainty due to conformational dynamics and the limited availability of high-confidence structural models [157]. Likewise, docking to LuxS or AI-2 receptor proteins (e.g., LsrB, LuxP) can be confounded by water-mediated contacts and the chemical complexity of AI-2 adduct recognition, features that are often simplified in high-throughput protocols [60].

Crucially, docking scores and predicted binding free energies should be interpreted as hypothesis-generating rather than confirmatory. Computational pipelines typically do not capture physiological determinants of activity, including bacterial permeability, efflux, and compound stability in biological media, and therefore cannot, on their own, establish QS target engagement. Accordingly, in silico prioritization is most valuable for rationalizing structure–activity trends (series-level consistency) and guiding experimental triage, whereas “top-scoring” single compounds require orthogonal validation. Minimal validation standards should include QS-specific reporter assays with stringent growth controls, analytical quantification of AIs where feasible, and direct receptor competition or enzymatic inhibition assays before candidates are classified as bona fide QS inhibitors.

### 5.3. From Phenotype to Target Engagement: Strength of Mechanistic Evidence for Phytochemical Quorum Sensing Inhibition

While the previous subsections document diverse phytochemical effects on QS-regulated phenotypes, a recurring limitation in this literature is that QS inhibition is often inferred from downstream outcomes rather than demonstrated by direct engagement of the QS machinery. Reduced biofilm biomass or virulence factor output, while suggestive, can arise through QS-independent mechanisms, including growth suppression, membrane perturbation, oxidative or acid stress, metabolic remodeling, and global transcriptional dysregulation. Accordingly, claims of specific “LuxI,” “LuxR,” “LuxS,” or “AI-2 receptor” targeting must be evaluated in light of the level of mechanistic validation provided. For the purposes of this review, the strength of mechanistic evidence can be organized through an operational hierarchy:

- Direct Target Engagement (Highest Confidence): This tier includes inhibition of purified synthases (e.g., LuxI, LuxS) or receptor binding/competition assays (e.g., LuxR-type proteins, AgrC, LsrB/LuxP). Ideally, such findings are supported by structure–activity relationships, orthogonal biophysical readouts (e.g., isothermal titration calorimetry, surface plasmon resonance), or genetic validation through mutagenesis and rescue experiments.
- QS-Proximal Functional Evidence (Moderate Confidence): This category encompasses reduced autoinducer concentrations quantified by analytical chemistry (e.g., LC–MS/MS), validated QS-specific reporter outputs with stringent growth-matched controls, or consistent modulation of defined QS regulons. While such evidence supports involvement of the QS pathway, it does not establish the precise molecular target.
- Phenotype-Only Evidence (Low Confidence): Studies reporting biofilm or virulence reduction without QS-specific readouts or appropriate growth controls fall into this tier. Such findings should be framed as correlative and hypothesis-generating rather than confirmatory of QS inhibition.

This evidence-tier perspective is particularly important for two scenarios: broadly acting phytochemicals with pleiotropic cellular effects, and conserved interspecies systems such as LuxS/AI-2, in which changes in biofilm formation or host interactions may arise through multiple overlapping pathways. Integrating direct target-engagement assays, autoinducer quantification, and QS genetic controls (e.g., QS-null mutants or pathway complementation) alongside standardized sub-MIC exposure frameworks will be essential for distinguishing bona fide QS inhibition from indirect physiological effects and for enabling mechanism-informed prioritization of phytochemical leads.

## 6. Challenges in Utilizing Phytochemicals as Quorum-Sensing Inhibitors

As discussed in the previous sections, several phytochemicals have been identified as potential QSIs, and numerous studies report that they attenuate QS-regulated phenotypes in bacterial pathogens. Despite this growing body of evidence, substantial gaps remain in our understanding of their safety, toxicological profiles, and translational feasibility, thereby hindering their practical application. Identifying phytochemicals that combine robust anti-QS activity with acceptable chemical stability, environmental compatibility, and host safety remains a key challenge.

Importantly, the limited translation of phytochemical QS inhibitors into clinical or industrial settings does not reflect a lack of biological activity. Rather, it stems from a convergence of mechanistic uncertainty, methodological variability, physicochemical constraints, and ecological complexity. As highlighted in a recent critical review by Alum et al. [28], multiple barriers, including incomplete mechanistic validation, inconsistent experimental frameworks, and insufficient consideration of safety and off-target effects, impede the progression of phytochemical QS inhibitors toward real-world applications. These challenges underscore the need for a more critical and systematic evaluation of phytochemicals as QS-modulating agents. Accordingly, this section examines the principal mechanistic, methodological, and translational limitations currently constraining the development of phytochemical-based QS inhibitors, with particular emphasis on the issues that must be addressed to enable their rational advancement as safe and effective anti-virulence strategies.

### 6.1. Variability and Lack of Standardization in Experimental Methodologies

A major limitation identified during the preparation of this review is the pronounced lack of methodological standardization across studies evaluating phytochemicals as QSIs. Although there is a broad consensus that many natural products can modulate QS-regulated phenotypes, conflicting and sometimes contradictory results are frequently reported. This issue is particularly evident for widely studied flavonoids and monoterpenoids—such as quercetin, naringenin, kaempferol, carvacrol, and L-carvone—whose reported QS-inhibitory efficacy varies substantially depending on experimental design, bacterial strain, and readout employed (Table 4 and Table 5).

In many studies, QS inhibition is inferred indirectly through reductions in biofilm formation, motility, or virulence factor production, without direct measurement of QS-specific endpoints such as autoinducer concentrations, LuxI/LuxS enzymatic activity, or signal–receptor interactions. As a result, it is often difficult to distinguish bona fide QS interference from secondary effects arising from metabolic stress, membrane perturbation, or growth retardation. This limitation is compounded by the frequent use of different reporter systems, growth conditions, and concentration ranges, which collectively hinder cross-study comparability and mechanistic interpretation.

The problem is further exacerbated by strain-dependent responses. Even within the same bacterial genus, phytochemical effects on QS can differ markedly depending on the strain’s genetic background, the specific QS circuit examined, and the biofilm’s maturation stage. Consequently, results obtained in one experimental system are not always transferable to others, limiting the generalizability of conclusions and complicating translational extrapolation.

An example of the above methodological fragmentation is particularly evident when comparing studies of structurally related monoterpenoids, such as carvacrol. While carvacrol is consistently reported to inhibit QS-associated phenotypes, the underlying mechanisms and experimental endpoints vary widely across studies. As summarized in Table 7, investigations differ with respect to bacterial species, QS readouts, analytical methods, and reporting of effective concentrations. For example, some studies directly quantify AHL levels using biosensor assays or LC–MS, whereas others infer QS inhibition solely from phenotypic suppression without measuring signal production or synthase activity.

Such discrepancies, including differences in assay type (biosensor vs. LC–MS vs. gene expression analysis), bacterial model (pathogenic vs. food-associated strains), QS endpoints (violacein, motility, biofilm formation, AHL quantification), and concentration units (mM, µg/mL, µL/mL),underscore the extent of methodological heterogeneity in the field. Without harmonized experimental frameworks, it remains challenging to determine whether observed QS inhibition reflects direct interference with LuxI-type AHL synthesis, indirect transcriptional regulation, or nonspecific physiological stress.

Collectively, these issues pose a major barrier to establishing reproducible potency metrics, comparing phytochemicals across studies, and drawing robust mechanistic conclusions. Addressing this challenge will require adopting standardized QS assays, including direct signal and enzyme measurements, and transparent reporting of experimental parameters. Without such rigor, the field risks overestimating the specificity and translational potential of phytochemical QS inhibitors.

The absence of standardized experimental frameworks remains a critical obstacle in quorum-sensing research. Without harmonized methodologies and direct mechanistic endpoints, it is not possible to reliably distinguish true QS inhibition from indirect or nonspecific effects, thereby limiting both mechanistic insight and translational progress. To improve cross-study comparability and reduce false attribution of “QS inhibition,” Table 8 summarizes commonly used QSI assays, their primary confounders, and the minimum validation criteria recommended for mechanistic interpretation and translational prioritization.

### 6.2. Questionable Selectivity of Phytochemicals and Potential Impact on Beneficial Bacteria

A fundamental, often underappreciated, limitation in developing phytochemicals as QSIs is their limited selectivity. QS systems are not exclusive to pathogenic bacteria but are widely conserved across pathogenic, commensal, and probiotic taxa. Core signaling components, particularly LuxS/AI-2 and, to a lesser extent, LuxI/LuxR-type systems, are shared among diverse microbial lifestyles. Consequently, phytochemicals designed to disrupt QS in pathogens may inadvertently interfere with QS-regulated functions in beneficial and symbiotic bacteria, raising concerns about unintended perturbation of the microbiome.

The potential impact of QSIs on commensal microbial communities remains poorly understood. QS plays a central role in coordinating interspecies interactions, metabolic cooperation, colonization, and host–microbe communication within complex microbial ecosystems. Broad interference with QS signaling therefore carries an inherent risk of dysbiosis, potentially undermining microbial homeostasis and host health. These risks are particularly relevant in the context of systemic or long-term QSI use, in which off-target effects on the resident microbiota may accumulate over time. We highlight LuxS/AI-2 first because of its universal interspecies role, then consider LuxI/AHL systems and broader mechanistic complications.

Concerns regarding selectivity are especially pronounced for LuxS/AI-2–mediated signaling. AI-2 functions as a universal interspecies communication molecule and regulates behaviors essential to microbial ecosystem stability, including biofilm formation, competitive fitness, and metabolic coordination. Experimental perturbation of LuxS/AI-2 signaling has been shown to alter probiotic traits: overexpression or AI-2 supplementation enhanced biofilm formation in bifidobacteria, whereas LuxS deletion in *L. rhamnosus* GG reduced biofilm, adhesion, and host colonization [90,93]. Thus, phytochemicals that inhibit LuxS activity or suppress AI-2 production may attenuate pathogen virulence while simultaneously destabilizing beneficial microbial communities.

However, the consequences of inhibiting QS in beneficial taxa remain inconclusive. A recent case study highlights this ecological ambiguity. Meng et al. [174] combined virtual screening, molecular dynamics/MM-PBSA, AI-2 reporter assays, RT-qPCR, and biofilm analyses to identify natural products (including mangiferin) that modulate LuxS activity in *Lactobacillus reuteri* 1-12. Notably, mangiferin reduced AI-2 reporter output yet increased probiotic biomass and biofilm integrity, illustrating that AI-2 pathway modulation by phytochemicals can produce context-dependent, potentially beneficial effects in commensal taxa. Taken together, these divergent outcomes point to two complicating factors: the pleiotropic activity of many phytochemicals and frequent indirect modulation of QS pathways. This example underscores that phenotype-only readouts cannot distinguish direct LuxS/LuxP/LsrB engagement from indirect metabolic or stress-related effects and highlights the need to evaluate candidate QSIs across representative commensal and pathogenic strains with targeted biochemical validation.

Although LuxI-dependent AHL signaling is most commonly associated with G^−^ pathogens, it is not exclusively linked to virulence. A subset of non-pathogenic and symbiotic G^−^ bacteria also employs AHL-mediated QS to regulate environmental adaptation, niche colonization, and interspecies interactions [65]. Broad inhibition of AHL biosynthesis or receptor function may therefore disrupt ecologically important processes in polymicrobial environments such as the gastrointestinal tract, oral cavity, and vaginal microbiome.

The pleiotropic nature of phytochemicals further complicates selectivity. Unlike conventional antibiotics, which target essential bacterial processes, many plant-derived compounds, including polyphenols, flavonoids, terpenoids, and phenolic acids, interact with multiple cellular targets. These compounds may affect enzymes, transcriptional regulators, membrane integrity, and redox-sensitive pathways, making it difficult to attribute observed QS inhibition to a single mechanism. This broad biological activity undermines the assumption that QS-targeting strategies are inherently microbiome-sparing.

Moreover, many phytochemicals modulate QS indirectly rather than by directly interfering with signal synthesis or reception. At sub-inhibitory concentrations, flavonoids and terpenoids can alter membrane fluidity, disrupt the proton motive force, or perturb intracellular redox balance, processes that influence QS signaling cascades downstream of canonical QS components. These indirect effects are unlikely to discriminate between pathogenic and beneficial bacteria, further eroding functional selectivity.

Importantly, most QS inhibition studies rely on monoculture models of beneficial bacteria and pathogen-centric endpoints, with limited attention to community-level effects or interspecies dynamics. As discussed in Section 3, QS serves fundamentally different roles in pathogenic versus beneficial bacteria, acting primarily as a virulence regulator in the former and as a homeostatic and cooperative mechanism in the latter. Disrupting these systems without accounting for ecological context may therefore lead to unintended outcomes, including reduced colonization resistance, compromised mucosal barrier function, or microbiome imbalance.

Furthermore, QS inhibition has been evaluated primarily in polymicrobial infection models, most commonly involving *P. aeruginosa* coexisting with *S. aureus* or fungal pathogens, where QS-targeted interventions can reduce virulence outputs, impair mixed-species biofilm development, and increase antibiotic susceptibility. These systems are intentionally designed to attenuate pathogen cooperation and persistence, rather than to assess impacts on beneficial microbial networks [175].

Although emerging work highlights that commensal microbiota possess diverse QS systems and may respond to both AI-1, AI-2, and peptide-based signals, direct experimental studies applying QS inhibitors or QQ enzymes to beneficial, commensal-dominated polymicrobial ecosystems remain scarce. A recent review on QS manipulation in commensal microbiota emphasizes that most current evidence derives from monoculture assays, pathogen-focused models, or conceptual proposals, with only early indications that QS/QQ could be leveraged for precision modulation of the gut microbiota [87]. Consequently, the field lacks robust in vivo or ex vivo studies demonstrating how QS inhibition alters the structure, stability, or functional outputs of beneficial polymicrobial consortia (e.g., gut, oral, or skin microbiota), leaving a major gap in understanding commensal ecosystem responses to QS disruption.

Taken together, these observations highlight a critical limitation of phytochemical QSIs: their biological activity is often insufficiently selective to ensure pathogen-specific targeting. Without a deeper understanding of QS network architecture, signal hierarchies, and context-dependent regulation across microbial communities, phytochemical modulation of QS risks replacing pathogen-driven dysbiosis with another form of microbial imbalance.

### 6.3. Physicochemical, Toxicological, and Pharmacokinetic Constraints Limiting the Translational Potential of Phytochemical QS Inhibitors

Despite the growing body of literature demonstrating phytochemical-mediated inhibition of QS, the translational potential of natural QSIs remains substantially constrained by physicochemical, toxicological, and pharmacokinetic limitations. While many QSIs exhibit robust anti-QS activity in vitro, their progression toward clinically viable anti-virulence agents has been impeded by inconsistent safety profiling, poor bioavailability, and inadequate validation in physiologically relevant models.

A fundamental limitation across the literature is the inconsistent reporting of mammalian cell toxicity. Although phytochemicals are often presumed to be inherently safe due to their natural origin, systematic toxicological evaluation is frequently lacking. In many studies, cytotoxicity is either not assessed or evaluated using a single cell line and limited concentration ranges, raising concerns regarding off-target effects and therapeutic windows. This issue is particularly relevant for microbial-derived QSIs such as surfactin and rhamnolipids, which, despite potent QS-inhibitory and antibiofilm activity, exhibit significant cytotoxicity at higher concentrations, thereby limiting their clinical applicability and host tissue compatibility [176].

In parallel, poor bioavailability and unfavorable pharmacokinetic profiles represent major barriers to systemic application of phytochemical QSIs. Many natural compounds, particularly polyphenols such as quercetin and curcumin, exhibit low aqueous solubility, rapid metabolic degradation, and short plasma half-lives, resulting in limited systemic exposure [177,178] despite strong in vitro QS inhibition. These properties severely restrict their effectiveness in vivo and complicate dose optimization. Consequently, QS inhibition observed under controlled laboratory conditions often fails to translate into consistent efficacy in animal infection models or clinically relevant settings.

The reliance on simplified in vitro experimental systems further exacerbates this translational gap. Most studies evaluating phytochemical QSIs employ planktonic cultures or static biofilm models that do not adequately recapitulate the complex physicochemical gradients, host immune pressures, and microbial community interactions present during human infections. As a result, compounds such as curcumin and allicin, which demonstrate marked inhibition of QS-regulated gene expression and biofilm formation in vitro, show inconsistent or attenuated efficacy in vivo, largely due to poor solubility, rapid metabolism, and limited tissue penetration [28,177].

Innovative drug delivery strategies, including nanoparticle encapsulation, liposomal formulations, and polymer-based carriers, have been proposed as potential solutions to overcome these limitations. Such approaches can enhance solubility, protect QSIs from premature metabolism, and improve tissue targeting. However, these strategies remain underexplored in the context of QS inhibition, and systematic comparative studies evaluating their impact on QS-specific endpoints, pharmacokinetics, and safety are still scarce [179].

Another unresolved challenge concerns interactions between QSIs and conventional antibiotics. Although several studies report synergistic or additive effects—such as enhanced antibiotic susceptibility and reduced biofilm tolerance when QSIs are combined with antimicrobial agents—other investigations document strain-dependent variability or no synergy at all. A representative example involves eugenol. Kong et al. [180] reported synergy between eugenol and colistin against colistin-resistant *P. aeruginosa* and *Klebsiella pneumoniae*, attributing the effect to membrane permeabilization that potentiates colistin. By contrast, Ashtiani et al. [181] examined eugenol’s impact on MexA and AcrA efflux pumps in *P. aeruginosa* and *E. coli*, reporting efflux inhibition and downregulation of pump expression as the mechanism increasing antibiotic susceptibility. Notably, both studies tested the same phytochemical but probed different mechanisms and contexts, illustrating how a single compound can produce divergent outcomes depending on species, antibiotic partner, assay, and experimental conditions. These discrepancies often reflect differences in experimental design (checkerboard/fractional inhibitory concentration index -FICi versus time-kill versus biofilm eradication assays), strain selection and growth phase, QSI and antibiotic concentrations, and the underlying QS circuitry. Consequently, there is a pressing need for standardized combination testing frameworks and mechanistically informed study designs that incorporate orthogonal assays, dose–response matrices, and representative in vivo or community models to resolve conflicting findings

Taken together, these constraints indicate that the limited clinical translation of phytochemical QSIs reflects not a lack of intrinsic biological activity but rather the convergence of suboptimal physicochemical properties, incomplete toxicological evaluation, insufficient pharmacokinetic optimization, and methodological limitations. To date, only a small number of QSIs have progressed beyond in vitro studies or preliminary animal models, and well-designed clinical trials assessing the safety, pharmacokinetics, and therapeutic efficacy of natural QS inhibitors in human infections remain notably lacking.

Despite their promise as sources of anti-virulence agents, the successful development of phytochemical QSIs into clinically or industrially viable interventions will require a shift from predominantly phenotype-driven screening toward mechanism-informed development strategies. In this context, rigorous toxicological profiling, pharmacokinetic refinement, advanced delivery systems, and validation in physiologically relevant infection models will be essential to bridge the gap between encouraging laboratory findings and effective real-world applications.

### 6.4. Potential for Resistance and Adaptive Responses to Quorum-Sensing Inhibitors

Targeting QS rather than bacterial viability assumes QSIs exert lower selective pressure, thereby reducing the risk of resistance. However, evidence indicates that bacteria can still adapt to bypass QS inhibition, thereby challenging the long-term effectiveness. In-host studies indicate resistance can develop but spreads more slowly than antibiotic resistance, emphasizing the need to study evolutionary dynamics in host models rather than just in vitro tests [182]. For example, the quorum-sensing model pathogen *P. aeruginosa* has been shown to develop resistance to QSIs in a synthetic medium containing adenosine as the sole carbon source [183]. Growth of *P. aeruginosa* on adenosine relies on the QS-regulated intracellular enzyme nucleoside hydrolase, and resistance in this context highlights how QS interference can directly affect bacterial fitness. It has therefore been suggested that the spread of resistance depends on whether QS influences fitness through regulation of private goods—products that benefit only the producer cell, such as nucleoside hydrolase—or public goods, such as extracellular proteases that support population-level growth on shared substrates [183].

Evidence for adaptive responses to QS inhibition is not limited to in vitro systems. Notably, the spread of resistance to quorum-sensing inhibition in populations of the QS model pathogen *V. campbellii* was monitored during up to 35 cycles of host infection and transmission, demonstrating that resistance can emerge and persist under biologically relevant conditions [184]. These observations underscore that QS-based strategies, while promising, are subject to evolutionary pressures similar to those acting on other antimicrobial interventions.

At the same time, accurate evaluation of QSI activity and avoidance of false conclusions regarding resistance require rigorous experimental validation to ensure that tested compounds do not impair bacterial growth. Minimal inhibitory concentration (MIC) assays must therefore be performed systematically, and QS-dependent phenotypic assays should be conducted strictly at sub-inhibitory concentrations [185]. At higher concentrations, many compounds classified as QSIs may exert conventional antibacterial effects by disrupting cell wall synthesis, nucleic acid replication, protein synthesis, or membrane integrity [186]. Failure to control for growth effects risks misattributing bacteriostatic or bactericidal activity to quorum-sensing inhibition, thereby confounding the interpretation of resistance and adaptation phenomena.

Importantly, even when growth effects are excluded, QSIs are not evolution-proof. Bacterial populations exposed to sustained QS inhibition can develop compensatory mechanisms that preserve communication, virulence, or fitness. One documented strategy involves activation of alternative or redundant QS pathways. For instance, *P. aeruginosa* can partially compensate for the inhibition of AHL-mediated signaling by upregulating secondary systems such as the *Pseudomonas* quinolone signal (PQS), thereby maintaining the regulation of virulence-associated traits [69,187].

Another well-characterized adaptive mechanism is the overexpression of multidrug efflux pumps, which actively export QSIs, thereby reducing their intracellular concentrations below effective thresholds [188]. Because many phytochemical QSIs are structurally similar to known efflux substrates, active export by multidrug efflux pumps can reduce intracellular QSI concentrations and limit sustained efficacy; phytochemicals have been reported both to inhibit and to be substrates of efflux systems, underscoring efflux as a key barrier to durable QSI activity [189]. In parallel, mutations in QS receptor proteins—such as LasR or RhlR in *P. aeruginosa*—can reduce QSI binding affinity while preserving responsiveness to native autoinducers, thereby restoring QS function under inhibitory pressure.

Beyond direct alterations to QS circuitry, metabolic bypass and regulatory rewiring further illustrate bacterial evolutionary plasticity. In some cases, bacteria upregulate alternative regulatory circuits or stress-response pathways that compensate for disrupted QS control, effectively decoupling virulence-associated phenotypes from canonical QS inputs [190]. Additionally, bacteria have been reported to produce decoy signaling molecules or enzymes that chemically modify or degrade QSIs, thereby reducing their functional availability [191].

Taken together, resistance to QSIs may develop more slowly than to antibiotics, but it is not negligible. Restoring group behaviors, such as biofilms and immune evasion, promotes adaptive responses during sustained QSI exposure. Long-term resistance studies are limited, creating a knowledge gap. While QSIs target communication rather than survival, resistance mechanisms, including efflux pumps, receptor mutations, pathway redundancy, and metabolic changes, are documented and must be addressed. Future efforts should focus on combination therapies, mechanistic validation, and long-term studies to ensure durable anti-virulence effects, as experimental evolution shows that mutations can restore group behaviors despite QSIs [183].

### 6.5. Translational and Regulatory Barriers

Despite substantial experimental evidence supporting the QSI activity of phytochemicals, their translation into clinically or industrially deployable anti-virulence agents remains limited. Beyond biological and mechanistic constraints, significant translational, regulatory, and manufacturing barriers continue to impede real-world implementation.

A major challenge arises from the intrinsic properties of natural products. Phytochemicals often exhibit batch-to-batch variability, complex and sometimes ill-defined chemical compositions, and limited scalability under good manufacturing practice (GMP) conditions. These factors complicate standardization, quality control, and reproducibility, requirements that are central to pharmaceutical development and regulatory approval [192].

Equally important, current regulatory frameworks are poorly suited to anti-virulence strategies. QSIs do not directly inhibit bacterial growth or viability, making their efficacy difficult to assess using conventional antimicrobial endpoints such as MIC or bacterial clearance. As a result, regulatory pathways for QS-targeting therapies remain underdeveloped, creating uncertainty in both preclinical evaluation and clinical trial design [193].

From a commercial perspective, investment in QSIs has been limited. Pharmaceutical development pipelines remain heavily biased toward bactericidal or bacteriostatic agents with well-defined regulatory precedents, and there is hesitancy to invest in non-traditional antimicrobial approaches whose clinical benefit may be context-dependent or adjunctive rather than standalone. Consequently, relatively few QSIs, particularly phytochemical-based agents, have progressed beyond in vitro or small-animal model studies [194].

Regulatory agencies such as the U.S. Food and Drug Administration (FDA) and the European Medicines Agency (EMA) will require clear guidance on efficacy endpoints, safety assessment, and the impact on the microbiome for QS-targeting therapies. Establishing such frameworks will likely necessitate new clinical trial designs that incorporate virulent attenuation, biofilm disruption, and synergy with conventional antibiotics as measurable outcomes. Large-scale, randomized controlled trials will be essential to define the therapeutic value of QSIs in human infections and to validate their proposed advantage of reduced resistance selection [195].

Addressing these challenges will require coordinated efforts across academia, industry, and regulatory bodies. Public–private partnerships, targeted funding mechanisms, and incentive structures may help bridge the gap between proof-of-concept studies and translational development. Ultimately, without rigorous mechanistic validation, standardized evaluation pipelines, and regulatory clarity, phytochemical QSIs are likely to remain confined to experimental settings despite their conceptual appeal.

### 6.6. Integrative Translational Assessment of Phytochemical QS Inhibition

To synthesize the mechanistic insights and translational challenges discussed above, Table 9 summarizes the key strengths, limitations, opportunities, and risks associated with phytochemical quorum-sensing inhibitors as anti-virulence agents. Collectively, these considerations underscore that the translational viability of QS-targeted phytochemicals is determined not by inhibitory potency alone, but by a balance between mechanistic specificity, pharmacokinetic feasibility, ecological selectivity, and safety within complex host–microbiome systems.

Importantly, many of the challenges identified are interdependent rather than isolated. For example, efforts to enhance QS-inhibitory potency through chemical modification or formulation may exacerbate off-target effects on commensal QS systems, whereas strategies aimed at preserving microbiome integrity may constrain efficacy against pathogenic populations. These trade-offs highlight the need for integrated evaluation frameworks that assess QS inhibition in biologically realistic contexts rather than simplified in vitro models. Building on this synthesis, the subsequent section outlines priority directions for future research, including rigorous mechanistic validation, standardized combination-testing paradigms, and microbiome-aware in vivo studies designed to advance QS-targeted phytochemicals toward clinically viable anti-virulence interventions.

## 7. Future Directions, Opportunities, and Recommendations

**Figure 2:**
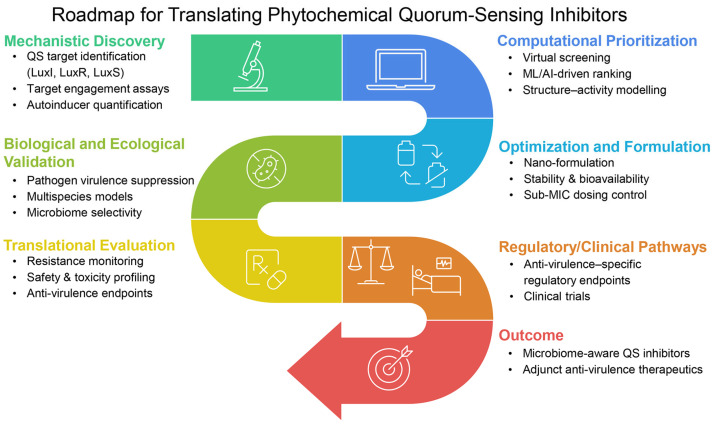
Translational roadmap for advancing phytochemical quorum-sensing inhibitors (QSIs) from discovery to application. The roadmap outlines the key scientific, technological, and regulatory steps required to translate phytochemical QS inhibitors into clinically or industrially viable anti-virulence strategies. Progress begins with mechanism-resolved target identification (e.g., LuxI, LuxR, LuxS, AI-2 signaling), followed by computational prioritization via virtual screening, machine learning, and structure–activity modeling to guide compound selection. Subsequent stages emphasize experimental validation, formulation and delivery optimization, and microbiome-aware evaluation in multispecies and host-relevant systems to minimize off-target effects on beneficial bacteria. The final phase highlights regulatory and translational considerations, including safety assessment, standardization, and combination strategies with existing antimicrobials. Collectively, the roadmap presents a coordinated, systems-level framework for overcoming current mechanistic, ecological, and translational barriers in developing phytochemical QS-targeted anti-virulence therapies.

Despite substantial progress in identifying phytochemicals that QS, the field remains at an early translational stage. Moving phytochemical-based QS inhibitors from proof-of-concept observations toward clinically or industrially viable anti-virulence strategies will require coordinated advances across multiple domains, including mechanistic target validation, compound prioritization, formulation and delivery optimization, microbiome-aware evaluation, and regulatory alignment. Addressing these interconnected challenges requires an integrated, forward-looking framework rather than isolated experimental efforts. Accordingly, this section outlines key future directions, opportunities, and strategic recommendations for advancing QS-targeted phytochemicals. These priorities are synthesized into a translational roadmap (Figure 2), which highlights the critical mechanistic, computational, ecological, and regulatory steps required to enable the rational development and real-world application of phytochemical QSIs.

### 7.1. Future Directions in Mechanism-Guided Quorum Sensing Inhibition

Future research should prioritize mechanism-based QS inhibition rather than phenotypic suppression alone. Although reductions in biofilm formation, motility, or virulence factor production remain valuable initial indicators, these outcomes should be systematically linked to defined molecular targets, such as LuxI, LuxR, LuxS, or downstream QS regulators, using integrated biochemical, genetic, and structural approaches. Direct measurement of autoinducer levels, enzyme kinetics, receptor binding, and target engagement should become standard practice to distinguish true QS interference from indirect metabolic or stress-related effects.

A parallel priority is the transition from simplified monoculture systems to biologically relevant models. Moving beyond static in vitro assays toward multispecies consortia, organoid-based infection models, and in vivo systems will be essential for capturing population-density effects, signal redundancy, and compensatory regulatory pathways that shape QS behavior under physiological conditions.

In addition, longitudinal studies are needed to monitor adaptive bacterial responses to sustained QS inhibition. Although QS inhibitors are often described as resistance-sparing, accumulating evidence shows that bacteria can rewire signaling networks, upregulate alternative QS pathways, or modify regulatory hierarchies over time. Understanding these adaptive trajectories is critical for designing robust, durable QS-targeted strategies.

### 7.2. Emerging Opportunities Enabled by Virtual Screening, Artificial Intelligence, and Advanced Technologies

Virtual screening (VS) has emerged as a powerful tool in modern drug discovery, enabling rapid, cost-effective identification of bioactive candidates from large chemical libraries. In the context of QS inhibition, VS, particularly when combined with molecular docking and molecular dynamics simulations—offers a scalable strategy to prioritize phytochemicals with high predicted affinity for QS targets such as LuxI, LuxR, and LuxS prior to experimental validation.

More recently, machine learning (ML) and artificial intelligence (AI) have begun to transform QS research by enabling pattern recognition, predictive modeling, and large-scale data integration. ML algorithms, including deep neural networks and random forest classifiers, can analyze complex QS datasets that include chemical features, docking scores, binding energetics, and phenotypic outputs to identify structure–activity relationships and predict QS inhibitory potential with increasing accuracy. AI-assisted workflows have already demonstrated utility in identifying high-affinity LasR antagonists and in prioritizing phytochemicals with favorable binding stability and pharmacological properties [196,197].

Beyond compound discovery, AI facilitates the integration of heterogeneous QS data into curated databases, enabling the mapping of complex QS networks across pathogens, commensals, and polymicrobial communities. Wu et al. [198] developed a ML–driven framework to systematically identify QS components across the human gut microbiota and reconstruct a comprehensive QS communication network (QSCN). By integrating manual curation, homology searches, and four ML classifiers, they expanded known QS entries to over 28,000 QS-related proteins across 818 gut microbial species and mapped nine major QS “languages,” including AI-2. This resource provides a foundational knowledge map for understanding interspecies signaling and designing QS-targeted therapeutic strategies.

In parallel, Tuan and Uyen [199] reframed QS systems as adaptive, closed-loop control architectures, demonstrating how ML-enhanced controllers enable robust pattern recognition, predictive scheduling of gene expression, and real-time feedback control in QS-regulated systems. Their work highlights improvements in biofilm suppression, toxin reduction, and antibiotic potentiation achieved by integrating sensing, inference, and control layers into unified frameworks, while emphasizing interpretability, safety-by-design principles, and standardized reporting.

AI-enabled QS analysis is also expanding beyond therapeutics into diagnostics and surveillance. Paper-based microfluidic assays coupled with ML classification have been used to identify bacterial species based on QS-driven aggregation dynamics under controlled flow conditions, enabling low-cost, field-deployable tools for potable water monitoring and microbial surveillance.

Collectively, integrating VS, ML, and AI offers a rational path toward mechanism-guided, scalable, and reproducible discovery of phytochemical QS inhibitors, reducing reliance on empirical screening and accelerating translational progress.

### 7.3. Key Recommendations for Translational and Microbiome-Aware Development of QS Inhibitors

Translating QS-targeted phytochemicals into clinically meaningful anti-virulence interventions requires a coordinated shift from isolated experiments to a cohesive translational research framework. Despite promising evidence that many natural compounds can suppress QS-regulated behaviors, including AI-2 signaling, biofilm formation, and virulence gene expression, the field remains fragmented by inconsistent methodologies, limited ecological validation, and insufficient attention to delivery, pharmacokinetics, and regulatory integration. Moreover, because QS pathways such as LuxS/AI-2 play central roles not only in pathogens but also in commensal and mutualistic members of the human microbiome, future development must balance therapeutic inhibition with ecological safety. A successful translational pathway, therefore, demands standardized assays, microbiome-aware evaluation, optimized delivery systems, rational combination strategies, and structured regulatory alignment. The following recommendations outline the essential steps needed to transform QS-focused phytochemicals from promising laboratory findings into robust, safe, and scalable anti-virulence tools.

- Standardization of Experimental Frameworks: QS inhibition studies should adopt harmonized protocols, including reliable MIC determination, validated QS-specific readouts, and standardized reporting of concentrations, growth conditions, and endpoints. Direct QS measurements should be prioritized over proxy phenotypes whenever possible.
- Microbiome-Aware Testing Pipelines: Given the central role of QS, particularly LuxS/AI-2 signaling, in beneficial bacteria, QS inhibitor development must incorporate microbiome-aware models. Multispecies consortia, gut-relevant systems, and host-associated models are essential for assessing off-target effects, dysbiosis risk, and ecological resilience.
- Optimization of Delivery and Pharmacokinetics: The therapeutic potential of many phytochemicals is limited by poor solubility, limited stability, and unfavorable pharmacokinetics. Systematic evaluation of formulation strategies, including nanoencapsulation, polymer-based carriers, and targeted delivery platforms, should be integrated early in development to maintain sub-inhibitory, anti-virulence concentrations over clinically relevant timeframes.
- Adjunctive Rather Than Replacement Strategies: QS inhibitors are most likely to succeed as adjunctive therapies, used in combination with antibiotics, bacteriophages, or immune-modulating agents. Rational combination studies using standardized synergy frameworks are required, alongside longitudinal monitoring of adaptive bacterial responses.
- Regulatory and Collaborative Alignment: Progress will depend on the establishment of clear regulatory pathways for anti-virulence agents, supported by consensus on efficacy endpoints, safety evaluation, and manufacturing standards. Public–private partnerships, shared QS databases, and interdisciplinary collaboration among microbiologists, chemists, data scientists, and clinicians will be critical to bridge the gap between discovery and application.

## 8. Conclusions

AR continues to undermine the effectiveness of conventional antimicrobials, prompting complementary strategies that attenuate pathogenic behaviors without directly targeting bacterial viability. In this context, QS remains a strategically attractive control layer because it coordinates virulence expression, biofilm maturation, and persistence programs that drive difficult-to-treat infections. In pathogen-focused studies, phytochemicals have emerged as a diverse source of QS modulators that suppress QS-regulated phenotypes by interfering with autoinducer availability and signaling perception. However, the central message of this review is that translational promise still exceeds mechanistic clarity. In many reports, QS inhibition is inferred from phenotypic changes rather than demonstrated by direct engagement of the target, making it difficult to distinguish bona fide QS interference from indirect physiological stress. Moreover, the conservation of QS systems across pathogens and commensals implies that efficacy cannot be evaluated in isolation from ecological selectivity, particularly for interspecies systems such as LuxS/AI-2, where microbiome-level effects may shape both safety and real-world performance.

Accordingly, advancing phytochemical QS inhibitors from proof-of-concept to application requires reframing success criteria from simple “phenotype suppression” to mechanism-verified, context-aware anti-virulence control. To facilitate this transition, a framework is proposed to connect discovery and implementation translation:

- Mechanism Specificity Hypothesis: Virulence attenuation will persist after controlling for growth and stress effects only when direct QS target engagement is demonstrated. This can be tested by pairing standard phenotypic assays with direct quantification of AI molecules (e.g., using LC–MS) and with target-binding or enzymatic assays at matched sub-MICs.
- Ecological Selectivity Hypothesis: Phytochemicals targeting species-specific LuxI/AHL signaling will exhibit greater pathogen selectivity than those perturbing the universal LuxS/AI-2 system, which is more likely to alter commensal community behaviors. Candidates should therefore be evaluated side by side in defined polymicrobial consortia (pathogen plus representative commensals), with community composition and host-relevant markers as primary endpoints.
- Combination Predictability Hypothesis: Reported “synergy” with antibiotics will be reproducible only under standardized dose–response matrices tied to defined mechanisms (e.g., efflux modulation or biofilm disruption). This requires harmonized checkerboard and time-kill workflows, pre-registered potency metrics, and orthogonal mechanistic readouts.

Ultimately, bridging the gap between in vitro discovery and clinical translation requires a shift toward rigorous, mechanism-resolved, and microbiome-aware evaluation. By systematically addressing mechanism specificity, ecological selectivity, and combination predictability, phytochemical QS inhibitors can advance from promising experimental leads to validated anti-virulence candidates to combat resistant pathogens.

## Figures and Tables

**Table 1:** Common quorum-sensing mechanisms and divergent biological outcomes in pathogenic and beneficial bacteria.

Feature	Pathogenic Bacteria *	Commensal/Symbiotic Bacteria *
Primary QS function	Coordination of virulence, biofilm formation, and resistance traits	Coordination of cooperation, stress tolerance, and colonization
QS systems involved	AHLs, AIPs, AI-2 (often hierarchical and virulence-linked)	AHLs, AIPs, AI-2 (often homeostatic and redundant)
Regulatory wiring	Often coupled to master virulence regulators	Integrated with metabolic and ecological regulation
Phenotypic output	Abrupt, threshold-dependent activation of pathogenic programs	Graded, adaptive responses
Effect of QS disruption	Strong attenuation of virulence and biofilms	Variable, often modest effects on fitness
Impact on viability	Viability preserved; pathogenicity reduced	Viability preserved
Evidence base	Extensive (in vitro, in vivo, clinical relevance)	Limited, mainly in vitro and strain-specific
Knowledge gaps	Translational application, resistance evolution	Microbiome-level and long-term effects

* Data were obtained from refs. [65,68,69].

**Table 2:** Quorum sensing–regulated mechanisms that indirectly contribute to antimicrobial resistance in pathogenic bacteria.

QS-Regulated Mechanism	Functional Role	Contribution to Antimicrobial Resistance	Refs.
Biofilm formation	QS coordinates biofilm initiation, maturation, and maintenance, leading to structured multicellular communities encased in a self-produced extracellular matrix	Limits antibiotic penetration, protects cells from immune clearance, and promotes long-term persistence	[81]
Horizontal gene transfer (HGT)	QS enhances conjugation, competence development, and cell–cell contact	Accelerates the dissemination of antibiotic resistance genes within and between bacterial populations	[84]
Efflux pump regulation	QS modulates the expression and activity of multidrug efflux systems	Reduces intracellular antibiotic accumulation, decreasing drug efficacy	[72,85]
Stress response and persistence	QS coordinates stress-adaptation pathways and promotes the formation of persister subpopulations	Enables transient tolerance to antibiotics without genetic resistance	[86]

**Table 3:** Quorum sensing–regulated mechanisms contributing to beneficial effects in symbiotic and probiotic bacteria.

QS-Regulated Mechanism	Functional Role in Beneficial Bacteria	Refs.
Spatial organization and niche adaptation	Regulation of microbial aggregation, density sensing, and spatial distribution in the gut	[68,88,89]
Adhesion and colonization	Promotion of mucosal adhesion, gastrointestinal transit, and biofilm formation	[90,92]
Biofilm formation (protective)	Formation of stable, non-pathogenic biofilms that support persistence and barrier function	[81,93]
Metabolic coordination	Regulation of iron uptake and metabolic cooperation among commensals	[94,104]
Host immune modulation	QS-mediated cross-kingdom signaling influencing inflammation and epithelial repair	[97,99]
Competitive exclusion of pathogens	Bacteriocin production and QS-regulated antagonism against invading microbes	[95]
Vaginal homeostasis	QS-associated biofilm formation and maintenance of a low-pH protective environment	[100,101,103]

**Table 5:** Phytochemicals targeting LuxS-dependent AI-2 biosynthesis.

Phytochemical/Extract	Phytochemical Class	Target Organism(s)	Evidence for LuxS/AI-2 Inhibition	Key QS-Regulated Effects	Ref.
Grape seed extract	Polyphenol-rich extract	*E. coli*	Reduced AI-2–dependent motility	↓ Flagellar motility, ↓ Shiga toxin	[131]
Oregano, rosemary, sage extracts	Phenolic diterpenes, polyphenols	*E. coli*	Strong AI-2 suppression (60–90%)	↓ Biofilm, ↓ swimming/swarming	[133]
Carnosol	Phenolic diterpene	*E. coli*	Docking studies In vitro LuxS inhibition	↓ AI-2 signaling, ↓ biofilm formation	[118]
Chlorogenic acid	Phenolic acid				
Curcumin	Polyphenol	*B. subtilis*, *P. aeruginosa*	In vitro AI-2 inhibition	↓ QS signaling, ↓ virulence	[134]
10-Undecenoic acid	Fatty acid derivative				
Carvacrol	Monoterpenoid	UPEC ^1^	↓ LuxS expression	↓ Biofilm ↓ motility	[132]
Cinnamaldehyde	Phenylpropanoid				
Eugenol	Phenylpropanoid				
Furosin, corilagin, ellagic acid (Triphala)	Hydrolysable tannins/ polyphenols	*H. pylori*	Docking studies MD ^2^ predicts LuxS stable binding	Predicted QS inhibition	[135]

^1^ UPEC: Uropathogenic *E. coli*; ^2^ MD: Molecular Dynamics; Symbol ↓ indicates reduction.

**Table 6:** Phytochemicals reported to modulate LuxR-family AHL receptors (LasR/RhlR/CviR) in Gram-negative bacteria (pathogen-focused evidence).

Phytochemical (Class)	Primary Receptor Target(s)	Model Organism(s)	Mechanistic Evidence for Receptor Targeting	QS-Linked Outcomes Reported	Ref.
Library of phytochemical hits (e.g., CID 5,281,647, 57,331,045, 5,281,672)	LasR	*P. aeruginosa* (in silico)	QSAR-guided virtual screening Docking and MD ^1^ studies Stable interactions with key LasR residues	Predicted LasR antagonism (virulence attenuation inferred)	[145]
Top ML-screened phytochemical hits (e.g., PubChem 3,795,981; 42,607,867; 6,971,066)	LasR	*P. aeruginosa* (in silico)	ML ^2^ prioritization, Docking and MD Studies Stable binding with canonical pocket residues	Predicted LasR antagonism (biofilm/virulence disruption inferred)	[146]
Flavonoid panel (naringenin, quercetin, apigenin, baicalein, etc.)	LasR and RhlR	*P. aeruginosa* receptor assays	Biochemical validation supporting allosteric/non-competitive inhibition (not necessarily pocket competition)	QS inhibition consistent with receptor-level blockade Downstream phenotypes reduced	[147]
Gingerol (Phenolic ketone– gingerol family)	LasR (proposed)	*P. aeruginosa*	Docking and phenotypic suppression consistent with LasR interference	Reduced virulence factor production and biofilm formation	[148]
Naringenin (Flavanone)	LasR	*P. aeruginosa*	Time-dependent competition model: effective when added early (before receptor activation), diminished when added late	Reduced QS-gene expression and virulence outputs when timed appropriately	[149]
Quercetin-rich onion extracts; quercetin aglycone; quercetin-3-glucoside Flavonol (and extract mixture)	CviR and LasR (docking-supported)	*C. vilaceum*, *P. aerginosa*, *S. macescens*	Docking and phenotype assays; aglycone often shows better receptor fit than glycoside	Reduced violacein and swarming Biofilm effects variable/limited at sub-MIC ^3^	[150]

^1^ MD: Molecular Dynamics; ^2^ ML: machine learning; ^3^ MIC: Minimum Inhibition Concentration.

**Table 7:** Methodological variability in representative studies evaluating carvacrol as a quorum-sensing inhibitor.

Study	Bacterial Model	QS System/Signal Examined	Primary QS Endpoints	Methodology	Key Findings	Mechanistic Resolution
Burt et al. [120]	*C. violaceum*	LuxI/LuxR (AHL)	Violacein, chitinase	Gene expression (*cviI*), phenotypic assays	Sub-MIC carvacrol downregulated *cviI* and reduced AHL-dependent phenotypes	Indirect evidence for LuxI suppression
Myszka et al. [158]	*P. fluorescens* KM121	AHL-mediated QS	AHL levels, motility, *flgA* expression, biofilm	Biosensor assay, LC–MS, qRT-PCR	~80% reduction in AHLs; suppressed motility and biofilm	Functional inhibition of AHL production; LuxI not directly assayed
Tapia-Rodríguez et al. [121]	*P. aeruginosa*	LasI/LasR, RhlI/RhlR	Violacein (biosensor), biofilm	Biosensor assay, biofilm quantification	Strong suppression of QS-dependent phenotypes	QS inhibition inferred; no direct LuxI measurement

**Table 8:** Common assays used to evaluate quorum-sensing inhibition and key limitations, confounders, and recommended validation steps.

Assay/Readout (Typical Use in QSI Studies)	Outcome (What the Assay Measures)	Major Limitations (Source of Heterogeneity)	Recommended Controls/Orthogonal Validation (Minimum Standard)	Refs.
MIC/sub-MIC definition (dose selection)	Growth inhibition threshold; defines “sub-inhibitory” range	“Sub-MIC” varies by strain/media/inoculum; QS phenotypes can change near growth-inhibitory zones; MIC often not re-determined under the QS assay conditions	Re-determine MIC under the same strain/media/inoculum conditions used for QS assays; report exact fraction (e.g., 1/8×, 1/4× MIC) and confirm no growth-rate change at test dose (growth curve and CFU)	[159,160,161]
Growth/viability controls (OD_600_, CFU, resazurin/XTT)	Fitness and viability; distinguishes QS effects from stress/growth inhibition	OD_600_ ≠ viability; metabolic dyes reflect activity (can change without killing); stress responses can suppress QS outputs indirectly	Include OD_600_ and CFU at matched time points and doses used for QS endpoints; if metabolic dyes are used, corroborate CFU; report inoculum, media, and time course	[161]
QS reporter strains/biosensors (pigment reporters; promoter fusions)	Reporter output linked to QS circuitry	Reporter signal can change with stress/redox/metabolism; pigment inhibition ≠ QS inhibition; strain background variability; signal uptake differences	Include growth controls and positive QSI control; verify reporter responsiveness (±exogenous AI); confirm with AI quantification (LC–MS/HPLC) and/or QS-regulon qPCR	[162,163]
Virulence-factor assays (elastase, protease, pyocyanin, rhamnolipids, hemolysins)	Downstream phenotypes often QS-regulated	Not uniquely QS-controlled; strongly influenced by growth phase, nutrient/iron status, and stress; endpoint-specific variability	Normalize to cell density and growth phase; include QS mutant or QS-inactivated control where feasible; support attribution with QS reporter/qPCR and/or AI quantification	[164]
Motility assays (swarming/swimming/twitching)	Behavioral outputs partly QS-regulated	Highly sensitive to agar %, plate drying, surfactants, viscosity, carbon source; growth inhibition falsely lowers motility	Standardize agar %, incubation, inoculum; confirm growth unaffected at dose; substantiate QS linkage with QS reporter/qPCR and/or AI measurements	[165]
Biofilm formation (crystal violet)	Total attached biomass (cells and matrix)	Confounds: growth suppression, detachment, matrix-only effects; high inter-lab variability; prevention ≠ eradication	Pair with planktonic growth control; add biofilm viability (CFU) or metabolic assay; report surface type, shear/flow, incubation time; test mature biofilms where relevant	[166]
Exogenous AI “rescue” (e.g., AHL/AI-2/AIP)	Whether the effect is upstream (signal) vs. downstream	Negative rescue not definitive (uptake/degradation/timing issues); AI form and timing critical	Specify timing (early vs. late), AI concentration/form; include positive rescue control; interpret alongside AI quantification and receptor/enzyme assays	[144,167]
Autoinducer quantification (LC–MS/HPLC; AI-2 activity reporters)	Signal abundance/activity in supernatants	AI-2 reporters are indirect and matrix-sensitive; analytical chemistry needs standards; QS is time-dependent	Report extraction/calibration/standards; sample multiple time points; normalize to growth; corroborate synthase inhibition or receptor assays when assigning targets	[168,169]
qPCR/transcriptomics of QS regulons	QS network gene-expression changes	Global stress can mimic QS downregulation; depends on marker selection and normalization	Use predefined QS-regulon markers and housekeeping controls; confirm no growth suppression confound; strengthen with AI quantification and/or target engagement assays	[170]
Purified enzyme assays (LuxI/LuxS inhibition)	Direct inhibition of AI synthesis machinery	In vitro inhibition may not translate intracellularly; aggregation/solubility artifacts; non-specific enzyme inhibition	Report enzyme/substrate conditions and IC_50_/kinetics; include solubility/aggregation controls; confirm concordant AI reduction in cells at matched sub-MIC exposures	[171,172]
Receptor binding/competition assays (LuxR-family; LsrB/LuxP; AgrC where feasible)	Direct receptor engagement	Membrane sensors are difficult; binding ≠ cellular efficacy	Report assay format and ligand controls; quantify competition/binding; connect to cellular QS readouts (reporter/qPCR) ± AI rescue to support causality	[44,106]
Docking/QSAR/MD (in silico prioritization)	Predicted binding pose/affinity (hypothesis generation)	Scores ignore permeability/efflux/stability; template uncertainty (esp. membrane sensors); over-interpretation risk	Present as hypothesis-generating; prioritize series-level SAR over single “top hits”; require wet-lab validation (binding/enzyme assays, AI quantification, QS reporters with growth controls)	[173]

Abbreviations: AHL: N-acyl homoserine lactone; AI: Autoinducer; AI-2: Autoinducer-2; AIP: Autoinducing peptide; CFU: Colony-forming units; HPLC: High-performance liquid chromatography;. IC_50_: Half-maximal inhibitory concentration; LC–MS: Liquid chromatography–mass spectrometry; LsrB: AI-2 binding protein of the Lsr transporter; LuxI: AHL synthase; LuxP: Periplasmic AI-2 binding protein; LuxR: AHL-responsive transcriptional regulator; LuxS: S-ribosylhomocysteinase (AI-2 synthase); MD: Molecular dynamics; MIC: Minimum inhibitory concentration; OD_600_: Optical density at 600 nm; qPCR: Quantitative polymerase chain reaction; QS: Quorum sensing; QSAR: Quantitative structure–activity relationship; XTT: 2,3-bis(2-methoxy-4-nitro-5-sulfophenyl)-2H-tetrazolium-5-carboxanilide.

**Table 9:** Translational strengths, limitations, opportunities, and risks of phytochemical quorum-sensing inhibitors.

Dimension	Key Points	Relevance to QS-Targeted Anti-Virulence Strategies
Strengths	Target virulence and biofilm formation without affecting bacterial viability Reduced selective pressure compared to bactericidal antibiotics Broad chemical diversity of phytochemicals enables interaction with multiple QS components Demonstrated synergy with conventional antibiotics	Supports anti-virulence paradigms and combination therapies aimed at mitigating antimicrobial resistance
Limitations	Poor solubility, stability, and bioavailability of many phytochemicals High concentrations are often required for QS inhibition in vitro Variability across experimental models and QS assays Limited in vivo and clinical validation	Constrains reproducibility and translational predictability
Opportunities	Nanocarriers and targeted delivery systems to improve pharmacokinetics Semi-synthetic optimization of phytochemical scaffolds Use as adjuvants to enhance antibiotic efficacy Precision QS modulation preserving beneficial microbiota	Enables rational design of next-generation QS inhibitors with improved safety and efficacy
Risks/Challenges	Redundancy and plasticity of QS networks enabling bypass mechanisms Potential off-target effects on commensal QS systems Incomplete understanding of long-term ecological consequences Regulatory uncertainty for anti-virulence therapeutics	Highlights the need for system-level evaluation and careful clinical translation

## Data Availability

No new data were created or analyzed in this study. Data sharing is not applicable to this article.
